# A Guided Size Exclusion Chromatography Demonstration for Teaching Polymer Separation and Characterization

**DOI:** 10.1021/acs.jchemed.6c00354

**Published:** 2026-09-07

**Authors:** Daniel Rivero, Nicole Paccha, Amrita Sarkar

**Affiliations:** † Department of Chemistry & Biochemistry, 8087 Montclair State University, Montclair, New Jersey 07043, United States; ‡ Department of Biology, 8087 Montclair State University, Montclair, New Jersey 07043, United States; § Sokol Institute for Pharmaceutical Life Sciences, 8087 Montclair State University, Montclair, New Jersey 07043, United States

**Keywords:** Second-year undergraduate students, Chromatography, Separation of polymer mixture, Hands-on learning, Instrument demonstration

## Abstract

Practical experience with modern analytical instruments is an essential component of undergraduate chemistry education, yet access to advanced instruments varies widely among institutions because of differences in available resources. Although size exclusion chromatography (SEC) is extensively used in polymer characterization in both academia and industry, undergraduate students at Montclair State University previously had limited opportunities to gain hands-on experience to this technique. To address this need, a demonstration using a multidetector-equipped Malvern Panalytical OMNISEC system was developed and incorporated into a second-year organic chemistry laboratory course during the 2025–2026 academic year. This activity engaged more than 300 undergraduate students representing chemistry, biochemistry, biology, psychology, marine science, and exercise science majors and was further extended to 8 undergraduate researchers and 6 K–12 participants through the Summer Polymer Research Program. Educational effectiveness was evaluated using worksheets, pre- and postdemonstration assessments, and anonymous students surveys. More than 95% of participants (*n* = 169) correctly answered the Fall 2025 postdemonstration worksheet questions, indicating a strong understanding of SEC concepts and instrument operation. In Summer 2026, students (*n* = 18) completed conceptually equivalent pre- and postdemonstration multiple-choice assessments. Mean scores increased from 7.72 ± 1.23 to 9.00 ± 1.03 (*p* < 0.001), demonstrating improved understanding following the demonstration. These findings show that a guided SEC demonstration provides an effective and adaptable approach for introducing advanced chromatographic instrumentation and polymer characterization to students from diverse scientific backgrounds.

## Graphical abstract

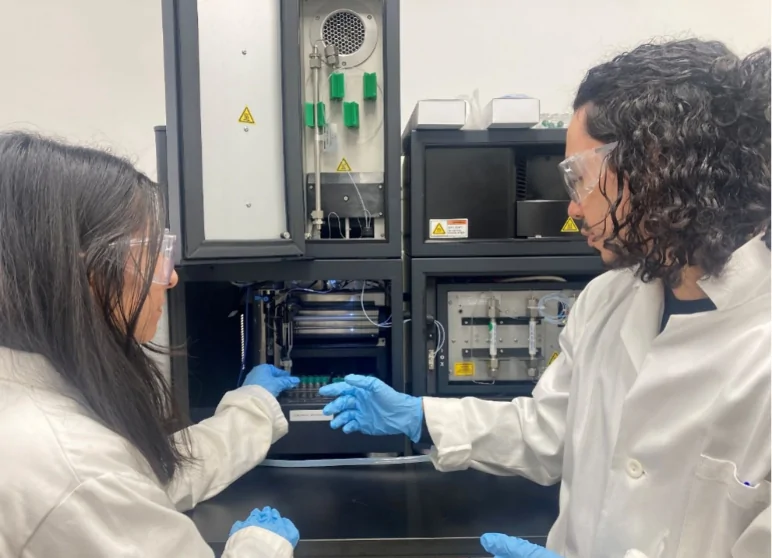

## Introduction

Proficiency in modern instrumentation and its applications is a fundamental component of chemistry education, as practicing chemists routinely employ a wide range of analytical tools to characterize and investigate chemical phenomena.^1^ According to survey data collected by the American Chemical Society (ACS) between 2011 and 2015, approximately 76% of newly graduated chemists entered careers in the chemical industry upon completion of their degrees.^2^ Prior studies^3,4^ have emphasized that exposure to modern instrumentation should be integrated into the undergraduate chemistry curriculum, particularly within organic and analytical courses. These studies further note that hands-on experience with instrumentation, an understanding of its operational principles, and the ability to relate instrument function to underlying chemical concepts are essential components of effective curriculum design aimed at improving students’ technical competencies. Therefore, a central instructional goal in undergraduate chemistry education should be to provide students with both theoretical and practical knowledge of modern instruments and to prepare them to apply these understanding in addressing scientific questions.^5,6^

Students enrolled in the undergraduate organic chemistry laboratory course in their second year at Montclair State University (MSU) are primarily introduced to liquid, gas, and thin-layer chromatography as part of the “*chromatography and separation*” category of instrumentation outlined in the ACS undergraduate guidelines.^7^ However, only a limited number of MSU students receive the opportunity to learn the theory and operation of size exclusion chromatography (SEC). SEC, also referred to as gel permeation chromatography (GPC), is a high-performance liquid chromatography (HPLC) technique that separates compounds based on molecular size. It is widely employed in various chemical applications, including the separation and molecular weight characterization of polymers, peptides, and proteins.^8–10^ SEC is a specialized form of liquid–liquid partitioning in which the stationary phase consists of a solvent-containing porous medium within the column, while the mobile phase is the solvent external to the column substrate particles.^10^ Analyte molecules larger than the pore size of the stationary phase are excluded from entering the pores and, consequently, elute first. Molecules of intermediate size, comparable to the pore dimensions partially penetrate the pores and elute subsequently, whereas smaller molecules diffuse freely into the pores and elute last. The conceptual separation mechanism is shown in Figure S1 in the Supporting Information (hereafter, SI).

Numerous recent technological advancements in bioimaging, smart coatings, food science, water purification, energy materials, and controlled drug delivery rely on SEC for the characterization of advanced polymer materials.^11–20^ Reflecting its broad industrial relevance, SEC has been identified as the 10th most frequently used instrument (out of 27 categories) in the chemical industry and as an instrument with which new chemists are expected to have practical experience.^3^ Although SEC has been incorporated into polymer-focused organic chemistry laboratories for senior undergraduates at MSU,^21^ opportunities for junior undergraduates to gain familiarity with this have been limited. Because SEC instruments are expensive, many undergraduate-focused institutions face significant barriers to offering students a direct, hands-on experience. As a result, practical exposure to SEC in introductory undergraduate teaching laboratories remains limited.

In early 2025, the Chemistry Department at MSU acquired and installed a new Malvern Panalytical OMNISEC multidetector-equipped SEC through a Department of Defense Equipment Grant (W911NF-24-1-0333) and was integrated into an introductory *Experimental Organic Chemistry Laboratory* course (CHEM 232) to provide students with foundational exposure to polymer separation and characterization. An engaging and accessible demonstration exercise was developed by using the OMNISEC to separate a polystyrene mixture. Although conducted as a guided demonstration, the small-group format combined with the simplicity of the experimental setup has enabled >300 students to independently perform one SEC experiment. While prior demonstrations, laboratory activities, and classroom lectures have introduced the concept of polymer and macromolecule separation, as well as the use of SEC, to a range of audiences including undergraduate, graduate, and high school students,^21–28^ to our knowledge, no previous reports have specifically combined SEC instrumentation, clear pedagogical design, and direct lower-level undergraduate student engagement with chromatographic data interpretation to teach the principles of polymer separation and characterization.

## Students’ Learning Goals

Assessments of students’ technical skills and instrument competence has consistently demonstrated that direct engagement with analytical instruments leads to greater mastery of practical laboratory techniques.^6^ Although several studies have examined students’ data interpretation, relatively few have discussed the implementation of hands-on learning with complex instrumentation in undergraduate teaching laboratories.^29,30^ The current SEC demonstration was designed to align with the instrumentation learning objectives outlined in the ACS Guidelines: *Undergraduate Professional Education in Chemistry*.^7^ Primary learning goals of the demonstration were 3-fold:

(i) To understand the fundamental principles of SEC and its relationship to macromolecular mixture separation.
(ii) To gain hands-on experience with the operation of the instrument and data analysis.
(iii) To appreciate the academic and industrial relevance of this technique.

The demonstration was implemented into two 2 h laboratory sessions, initially integrated into the CHEM 232 Laboratory course. CHEM 232 was selected for two reasons: (1) it is the first instrument-intensive laboratory course that students complete after limited exposure to instrumentation in general chemistry at MSU, and (2) it enrolls a large and diverse population of students majoring in chemistry, biochemistry, biology, molecular biology, psychology, exercise science, and marine biology. The demonstration was conducted with 234 students in fall 2025 as well as 50 students across summer 2025 and 2026 terms. Furthermore, it was implemented in CHEM 130: Fundamentals of Organic Chemistry during spring 2026 with 20 nutrition majors. The activity was further extended to 8 undergraduate research students and 6 K–12 participants enrolled in the Summer Polymer Research Program in 2025 and 2026.

## Experimental Overview

The demonstration conducted during Fall 2025, over a two-week period, across 11 scheduled lab sessions is described here. A total of 234 students from multiple majors, organized into 114 two-student teams, is summarized in Figure S4, which provides the STEM major distribution of the participating cohort. The activity was designed as a two-scheduled 2 h student-facing laboratory instructional module embedded within a longer instructor-managed analytical workflow, rather than as a complete 4-h sample preparation-to-analysis experiment. Before the lab began, polystyrene (PS) stock solution was prepared by two undergraduate research assistants. During the first 2-h session, each participating student team filtered the premixed polymer solution into autosampler vials, loaded them onto the instrument, and completed the sample-entry and sequence-setup procedures in the OMNISEC software. Following this, the SEC instrument completed an automated analytical sequence requiring ∼5 h. The instrument therefore continued operating beyond the scheduled laboratory period, and student attendance was not required during this time. During the second 2-h session, students were provided with chromatographic results and completed guided data processing, interpretation, and discussion activities; Table S1 in SI summarizes the whole activity. Institutions without the ability to operate the instrument outside a standard laboratory period could adapt this activity by using instructor-generated data, previously collected chromatograms, or a reduced sample sequence.

### Day One Activity

#### Pre-Lab Session and Instrument Demonstration

The session began with a 20 min prelaboratory lecture delivered by the instructor, introducing the fundamental SEC principle and related theoretical concepts, including number-average (*M*_n_) and weight-average (*M*_w_) molecular weights, dispersity (*Đ*), intrinsic viscosity (IV), hydrodynamic radius (*R*_h_), etc., detailed in the Section S1: Students Handout, SI. Then, students observed a live demonstration of the OMNISEC instrument and were introduced to its main components, detailed in Section S2: OMNISEC System Live Demonstration, SI. Students were reminded that, unlike HPLC where separation primarily depends on the surface area and properties of the packing material, SEC separation relies solely on the accessibility of the pores based on analyte size. Students were introduced to its four detectors (Figure S5), including concentration, absorption, viscometer, and light scattering, that operate simultaneously for comprehensive characterization. Students were informed that a suitably configured HPLC equipped with either a differential refractive index (DRI) or a UV detector, in combination with SEC columns and appropriate data analysis software, can be used for SEC measurements to obtain relative molecular weight values through conventional calibration. In contrast, parameters such as IV, *R*_h_, and radius of gyration (*R*_g_) require the incorporation of viscometer and/or light scattering detectors. It was further emphasized that SEC systems differ in both configuration and analytical capabilities and, therefore, do not necessarily provide equivalent data outputs.

#### Sample Preparation or Methods and Measurement

Following the instrument demonstration, three commercially purchased PS standards (*M*_w,PS1_ = 12,800 g mol^–1^, *M*_w,PS2_ = 245,000 g mol^–1^, and *M*_w,PS3_ = 4,760,000 g mol^–1^) mixed in equal weights at 1 mg mL^–1^ in tetrahydrofuran (THF) were provided to the students. Each team filtered 1 mL of the premixed PS solution through a 0.2 μm PTFE syringe filter, loaded 100 μL into autosampler, and arranged samples in sequence via OMNISEC software, following step-by-step instructions provided in Section S3: OMNISEC Software Operation, SI, containing the procedural guide and workflow screenshots (Figures S6–S9). No unexpected or unusually high safety hazards were encountered. Due to the spatial limitations of the laboratory housing the instrument, a maximum of 4 groups (8 students) could be accommodated at a time. Consequently, the instrument demonstration for 10–11 groups in a single lab section was completed in 2 h. Students observed the sample loading and initiation of the measurement sequence but were not required to remain for the entire duration.

### Day Two Activity

#### Data Analysis

Each team received a printed copy of their corresponding sample’s chromatogram (Figure 1 and Figure 2) and was briefly guided through the data acquisition and processing steps (Section S3, SI includes the full workflow and annotated screenshots, Figures S10–S14). Students received a complete set of information about their samples from a single injection using a triple advanced detector system (RI, Viscometer and Light Scattering), *vide infra*. Before demonstration, conventional and universal calibration curves were prepared by the instructor using PS standards (Section S6: Instructor’s Notes, SI, Figures S18–S19). The data analysis method involves calculating sample concentration based on the RI increment (d*n*/d*c*) value (for PS in THF, a d*n*/d*c* value of 0.185 mL g^–1^) that was reinforced during the data analysis. Under instructor supervision, each team analyzed their chromatogram, completed the postlab assignment, and participated in an evaluation survey, as discussed below. Most injections yielded successful chromatograms, with only 7 teams showing no signals (Figure S15), likely due to injection failure or reduced sample volume from THF evaporation during overnight runs.

**Figure 1:**
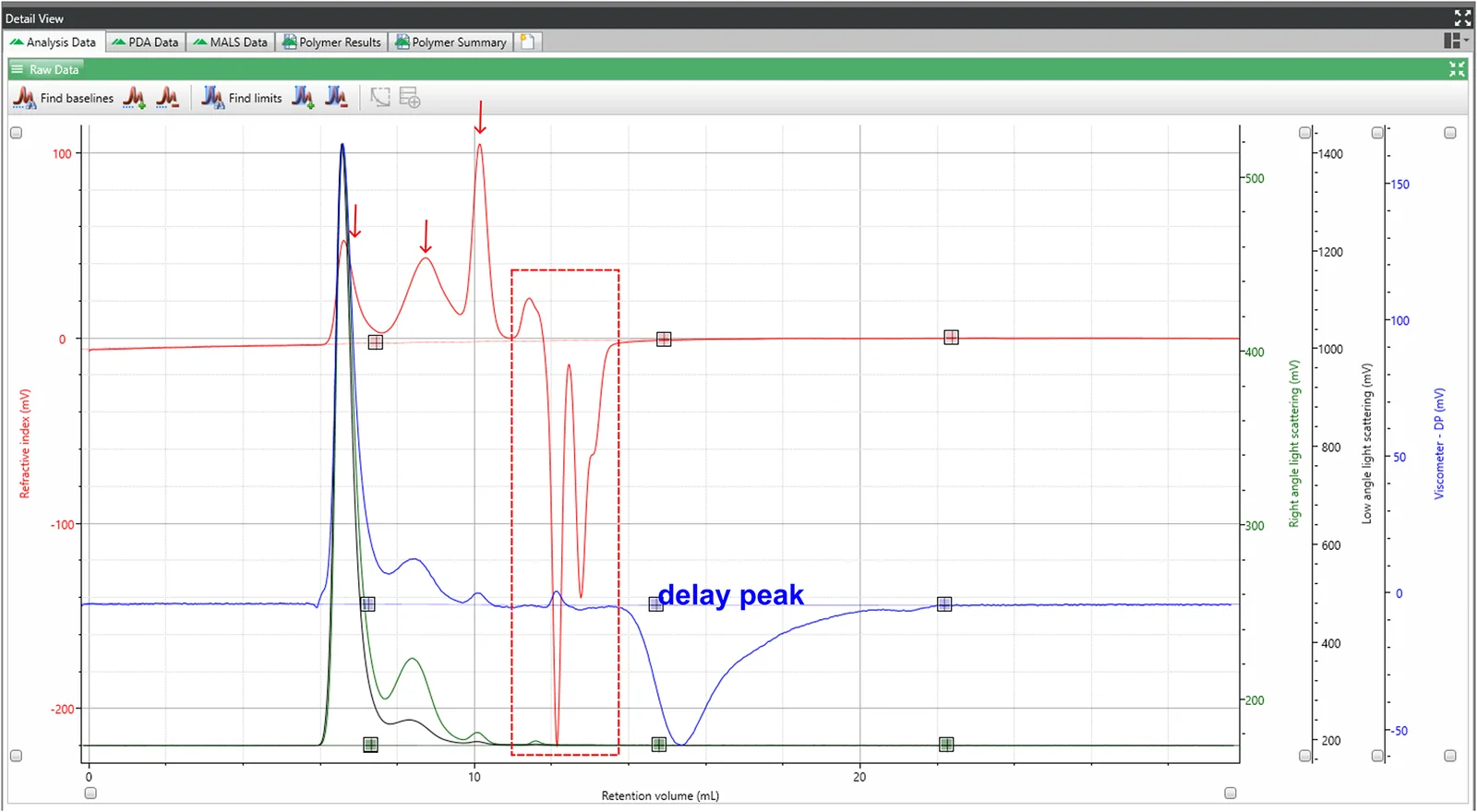
Full-range SEC chromatogram for PS mixtures recorded using multiple detectors: refractive-index (red), right-angle light-scattering (green), low-angle light-scattering (black), and viscometer (blue). The three sharp, well-resolved PS peaks are indicated by red arrows and eluted within 11 mL with minimal peak overlap. The negative RI response at ∼12–14 mL, enclosed by the red dashed box, is attributed to a refractive-index mismatch between the injected sample and the mobile phase, potentially arising from low molecular weight additives or stabilizers. The broad negative viscometer response at ∼15–20 mL is the detector-delay peak associated with the instrument’s delay volume and transient pressure imbalance in the capillary bridge.

**Figure 2:**
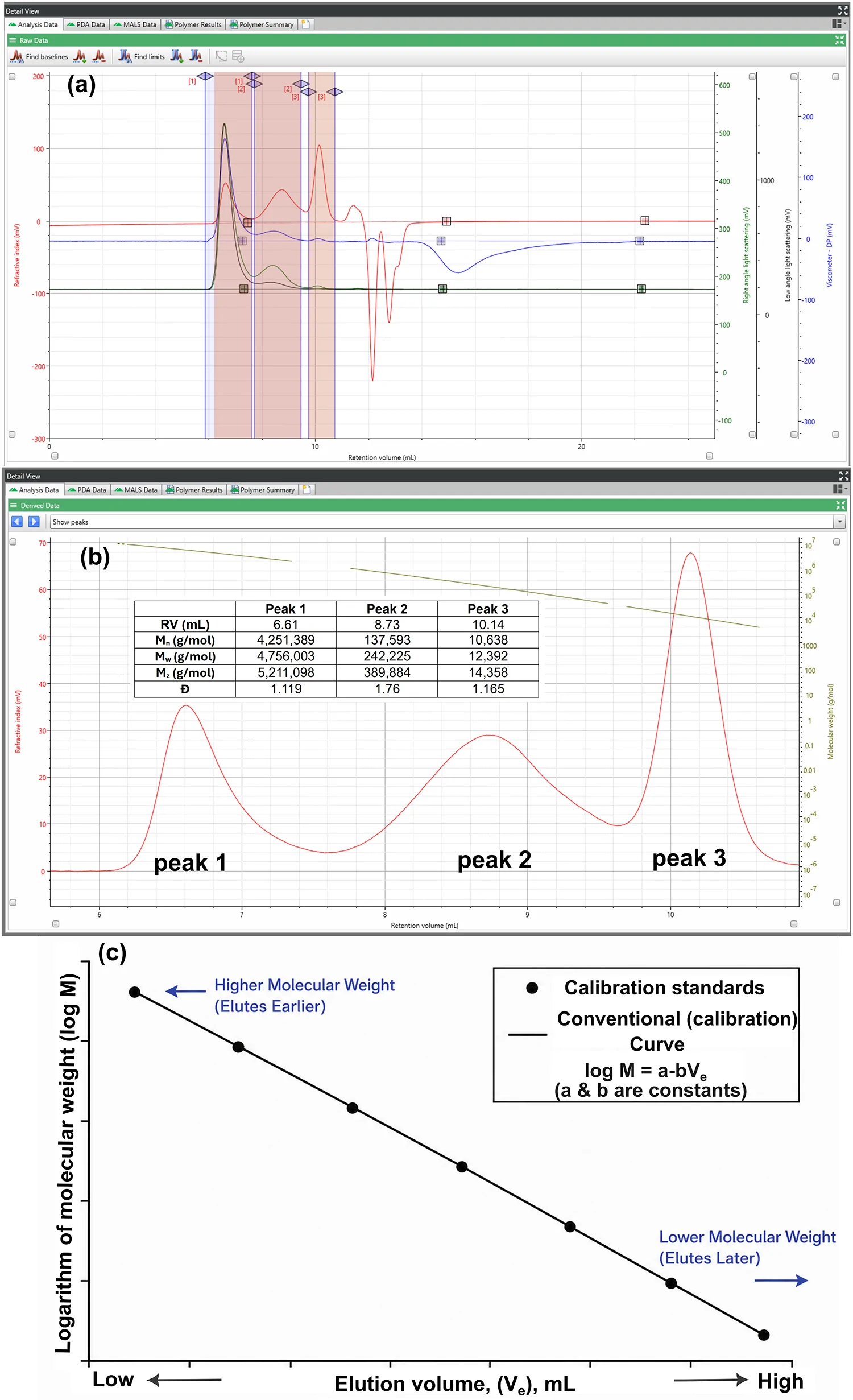
SEC data analysis of PS mixture using OMNISEC software. (a) Analyzed multidetector chromatogram showing the selected integration regions for the three PS components. (b) Derived RI chromatogram after baseline correction and individual peak resolution. Only the RI detector channel is displayed to improve clarity and avoid overlapping with the other detector signals. Peaks 1–3 correspond to the three resolved PS components, and the inset summarizes the calculated molecular weight parameters for each peak. (c) Schematic representation of a conventional SEC calibration curve in which the logarithm of molecular weight is plotted against elution volume. Higher molecular weight polymers elute earlier at lower elution volumes, whereas lower molecular weight polymers elute later at higher elution volumes.

## Results and Discussion

During day 2, each team discussed their collected chromatograms, as described below.

### Assessment of Separation Success

Students focused on three key features of their chromatograms, shown in Figure 1. First, the PS mixture was resolved into three sharp, symmetric, and positive peaks (indicated by red arrows), which were consistently detected by all three detectors (UV detector was excluded from this analysis to reduce measurement time). Three components, corresponding to distinctly different molecular sizes, were well resolved with no significant peak overlap. Complete separation was achieved within 11 min, noting that retention time and retention volume are equivalent at a mobile phase flow rate of 1 mL min^–1^. Second, a negative peak was observed in the RI trace at ∼12–14 mL (highlighted by red dotted box). Such peaks occur when RI of a component of the injected sample differs from that of the pure mobile phase, often due to stabilizers or other low molecular-weight additives present in the sample and/or solvent. Students understood that the resulting RI mismatch causes the solution in the sample side of the RI detector to exhibit a lower refractive index than the mobile-phase baseline, producing a negative deflection in the RI signal. Lastly, students observed a negative peak at ∼15–20 mL in the viscometer detector signal (delay peak), which is typically caused by the detector’s intrinsic delay volume and transient pressure imbalances in the capillary bridge.

### Linking Chromatographic Results to SEC Principles

Each team summarized key features of their chromatogram utilizing OMNISEC software, shown in Figure 2. Students were reminded again that molecules larger than the column pores elute earlier, while smaller molecules penetrate the pores and elute later. Students were subsequently asked to apply this conceptual understanding to their derived chromatograms by identifying peaks corresponding to three PS standards with distinct *M*_w_ and *Đ* provided in Day One. Most participants successfully identified Peak 1 as PS3, which eluted at 6.61 mL and represented the highest molar mass polymer (given *M*_w,PS3_ = 4,760,000 g mol^–1^). They also correctly recognized Peaks 2 and 3 as PS2 and PS1, eluting at 8.73 and 10.14 mL, corresponding to the intermediate (given *M*_w,PS2_ = 245,000 g mol^–1^) and lowest (given *M*_w,PS1_ = 12,800 g mol^–1^) molar mass standards, respectively. Students’ attention was further directed to the molecular weight trace in the Derived Data window, which intersects the RI chromatogram (Figure 2b), reinforcing its relationships with elution volume. Finally, students evaluated the numerical values for each PS obtained (using the conventional calibration method, Figure S18) with a representative student team’s results inscribed in Figure 2b. One representative team reported measured *M*_w_ values of 12.4 kg mol^–1^, 242 kg mol^–1^, and 4.76 million g mol^–1^ for PS1, PS2, and PS3, respectively, with corresponding dispersities (*Đ*) of 1.16, 1.76, and 1.12. The representative team commented that the mixture contains two narrowly dispersed PS1 and PS3 and one broadly distributed PS2. Students realized that SEC with concentration sensitive detectors (e.g., RI) yields relative molecular weight values, as that rely on calibration with standards and are influenced by column efficiency and instrument performance. The majority of the students’ teams observed good agreement between their measured molecular weights, relative to PS calibration standards, and the values provided on Day One. Students were cautioned that ultrahigh molecular weight polymers (e.g., PS1 = 4.76 × 10^6^ g mol^–1^) are shear-sensitive and may undergo chain scission under typical SEC conditions, resulting in apparent degradation at elevated flow rates. They were further encouraged to critically evaluate the suitability of an injection concentration of 1 mg mL^–1^ for future SEC analysis of such polymers, recognizing that the higher concentration can exacerbate shear-induced degradation and contribute to nonideal separation behavior.

The instructor presented overlays of randomly selected 10 measurements from Fall 2025 and 24 measurements from Summer 2026 laboratories, along with accompanying statistical analysis, allowing students to assess agreement among measurements and the overall precision of the data set (Figures S16–S17, Table S3). Students also examined the other derived results including IV and *R*_h_ (using the universal calibration method and light scattering detector) from their individual chromatogram (Table S2). Although IV comparisons were not directly relevant to this data set since all three polymers were polystyrene, students recognized how differences in IV can be used to infer structural features of different polymers with similar molar masses. Specifically, a combination of low IV and low *R*_h_ suggests a more compact architecture (e.g., hyperbranched polymer), whereas higher IV and larger *R*_h_ values are indicative of a more extended structure (e.g., linear polymer).^31^ One representative student datasheet showed that IV and *R*_h_ increased from PS1 to PS3 and ranged from 0.05 to 7.40 dLg^–1^ and 2.3 to 82 nm, respectively, consistent with the increasing molar masses of the standards.^32^

## Hazards and Safety

The OMNISEC operation does not present unusual hazards. However, THF is a flammable peroxide-forming solvent (stabilized with butylated hydroxytoluene, BHT) with acute toxicity and the potential to cause damage to the kidneys, liver, and eyes. Therefore, the use of appropriate personal protective equipment including safety goggles, gloves, and laboratory coats is strongly recommended throughout this activity. THF waste was collected in properly labeled, sealed, and designated organic solvent waste containers, kept segregated from incompatible chemicals, and disposed in accordance with institutional health, safety, and hazardous waste protocols.

## Assessment of Learning Outcomes

During Fall 2025, student learning was evaluated using postdemonstration worksheets (Section S5, SI) and anonymous surveys (Figure 3). Over 95% of the responses to the worksheet were correct, indicating a high level of postactivity performance. Anonymous surveys administered during the Fall 2025 activity indicated that the demonstration was successful. One student commented, *“the demonstration was interesting because it helped me actually see how molecules of different sizes separate during chromatography. It made more sense once I saw the graph and how retention volume is related to molecular weights.”* Another wrote, *“I found it very useful session that we were given a thorough explanation of what each chamber in the machinery was utilized for.”* One comment was “*I found it useful when we were reading the graph to see our end outcome”*. To quantitatively evaluate changes in student performance, a pre- and postdemonstration assessment was conducted during the Summer 2026 offering of CHEM 232. The participating cohort included 20 students: 18 completed both assessments, while 2 students completed only the postdemonstration assessment. Therefore, the analysis of changes in performance was based on the 18 matched pre- and postassessment scores. They completed conceptually equivalent 10 question multiple-choice assessments before and after the demonstration (Section S5, SI) with both scored out of 10 points. The mean score increased from 7.72 ± 1.23 to 9.00 ± 1.03. A paired-samples *t* test indicated that this increase was statistically significant, *t*(17) = 5.05, *p* < 0.001, with a Cohen’s *d*_*z*_ effect size of 1.19. Together, the qualitative feedback from Fall 2025 and the quantitative pre/post-assessment from Summer 2026 show that the guided SEC demonstration effectively enhances students’ understanding of SEC principles, instrument operation, and chromatographic data interpretation. The MSU Institutional Review Board (IRB) determined that this project does not constitute human subjects research and does not require IRB approval. However, we acknowledge that the assessment was conducted with a relatively small cohort of students. Future implementations involving larger and more discipline-diverse cohorts and delayed assessments will help evaluate the generalizability and persistence of the observed learning gains.

**Figure 3:**
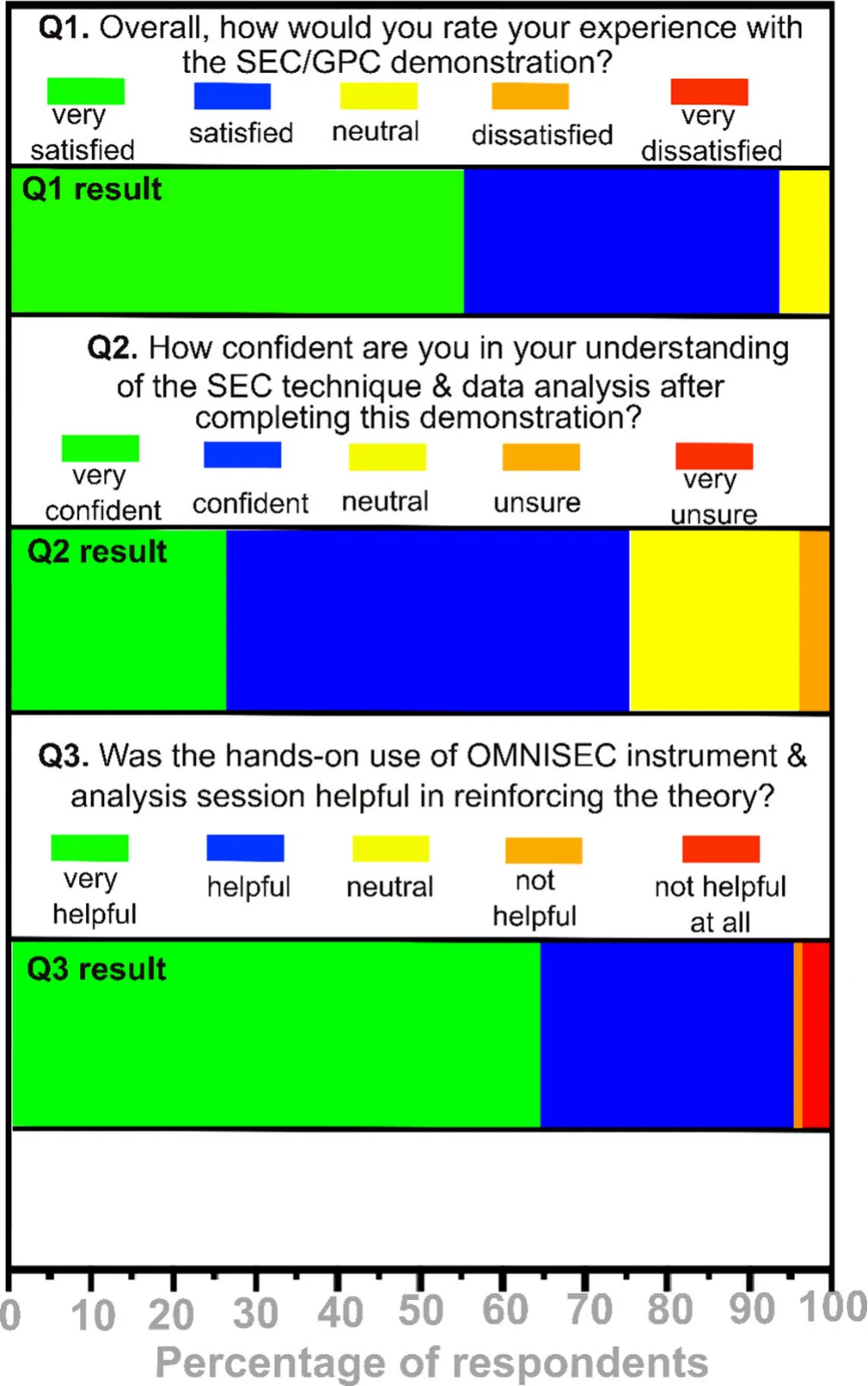
Student survey results following the Fall 2025 SEC/GPC demonstration (*n* = 169 undergraduate students). The stacked horizontal bars show the percentage of respondents addressing Q1, Q2, and Q3. Response categories are identified by the color-coded legends above each bar.

## Conclusion

With the increasing academic and industrial importance of polymer science, the OMNISEC demonstration described herein provides an effective introduction to SEC to our lower-level undergraduate students. By integration of a state-of-the-art polymer separation and characterization technique into teaching laboratories at MSU, this activity aligns with the ACS guidelines for undergraduate chemistry education and supports the development of instrumental analysis competencies. The learning objectives are scaffolded to accommodate a broad range of student-experience levels. This activity is adaptable to advanced organic and polymer laboratory courses through inquiry-based and research-oriented projects where students will engage in advanced data interpretation, including the use of multiple detectors to determine the absolute molecular weight, IV, and *R*_h_ of linear and hyperbranched polymers. Overall, this demonstration provides a flexible and scalable framework for incorporating contemporary polymer characterization techniques into undergraduate chemistry curricula and better prepares students for research and industry careers.

## Supplementary Material





## Notes

Student and instructor materials, including SEC theory, OMNISEC instrumentation and software-operation instructions, representative student-generated data, pre- and postdemonstration worksheets, postlab worksheet, instructor notes, and conventional and universal calibration information (PDF; DOCX)
