# Supplementary material for: A Guided Size Exclusion Chromatography Demonstration for Teaching Polymer Separation and Characterization
Source: J Chem Educ. 2026 Sep 7;103(10):6170–6. doi: 10.1021/acs.jchemed.6c00354 (PMC13673999; doi:10.1021/acs.jchemed.6c00354)
Supplement: Supplementary file 1 [file ed6c00354_si_001.pdf]

Supporting Information

A Guided Size Exclusion Chromatography Demonstration for Teaching Polymer Separation and Characterization

Daniel Rivero, [a] Nicole Paccha, [b] Amrita Sarkar *, [a, c]
[a]*Department of Chemistry & Biochemistry, Montclair State University, Montclair, NJ 07043, USA.*
[b]*Department of Biology, Montclair State University, Montclair, NJ 07043, USA.*
[c]*Sokol Institute for Pharmaceutical Life Sciences, Montclair State University, Montclair, NJ 07043, USA.*
**Corresponding author: sarkara@montclair.edu*

Section S1. Students Handout……………………………………………………………………..S1-S5
Section S2. OMNISEC Live Demonstration…………………………………………………… …...S5-S7
Section S3. OMNISEC Software Operation……………………………………………………...S8-S14
Section S4: Students Data……………………………………………………………………...S15-S17
Section S5: Pre- &Post-demonstration Worksheet……………………………………………….S18-S20
Section S6: Instructor's Note…………………………………………………………………….S21-S23
Section S7: References…………………………………………………………………………..…S23

Section S1: Students Handout

Materials

Narrow and broad calibration standards polystyrene were purchased from Malvern Panalytical Inc. HPLC grade methanol (99.9%, Spectrum™ Chemical), water (ultrapure, Thermo Scientific Chemicals) and tetrahydrofuran (99.9%, stabilized with BHT, Spectrum™ Chemical) were purchased and used as received without further purification.

Theory of Size Exclusion Chromatography (SEC)

The separation mechanism in size exclusion chromatography (SEC), also known as gel permeation chromatography (GPC), is governed exclusively by the relative molecular size of the analytes compared to the pore size of the stationary phase (**Figure S1**). SEC represents a specialized form of liquid-liquid partitioning in which the stationary phase consists of a solvent-containing porous medium within the column, while the mobile phase is the solvent external to the column substrate particles. [1-3] SEC employs a stationary phase composed of a column packed with porous beads whose pore sizes are, on average, comparable to the molecular sizes of the analytes being separated. When a mixture of analytes passes through the column, larger molecules are unable to enter most of the pores in the packing material due to size exclusion and therefore elute first. Molecules of intermediate size partially penetrate the pores, spending a moderate amount of time within the stationary phase and eluting subsequently. In contrast, smaller molecules can access a greater proportion of the pore volume, resulting in longer retention times and elute at higher retention volumes. This size-dependent separation mechanism enables the effective separation or fractionation of mixtures containing macromolecules of varying molecular size, with the largest species eluting first and the smallest eluting last.

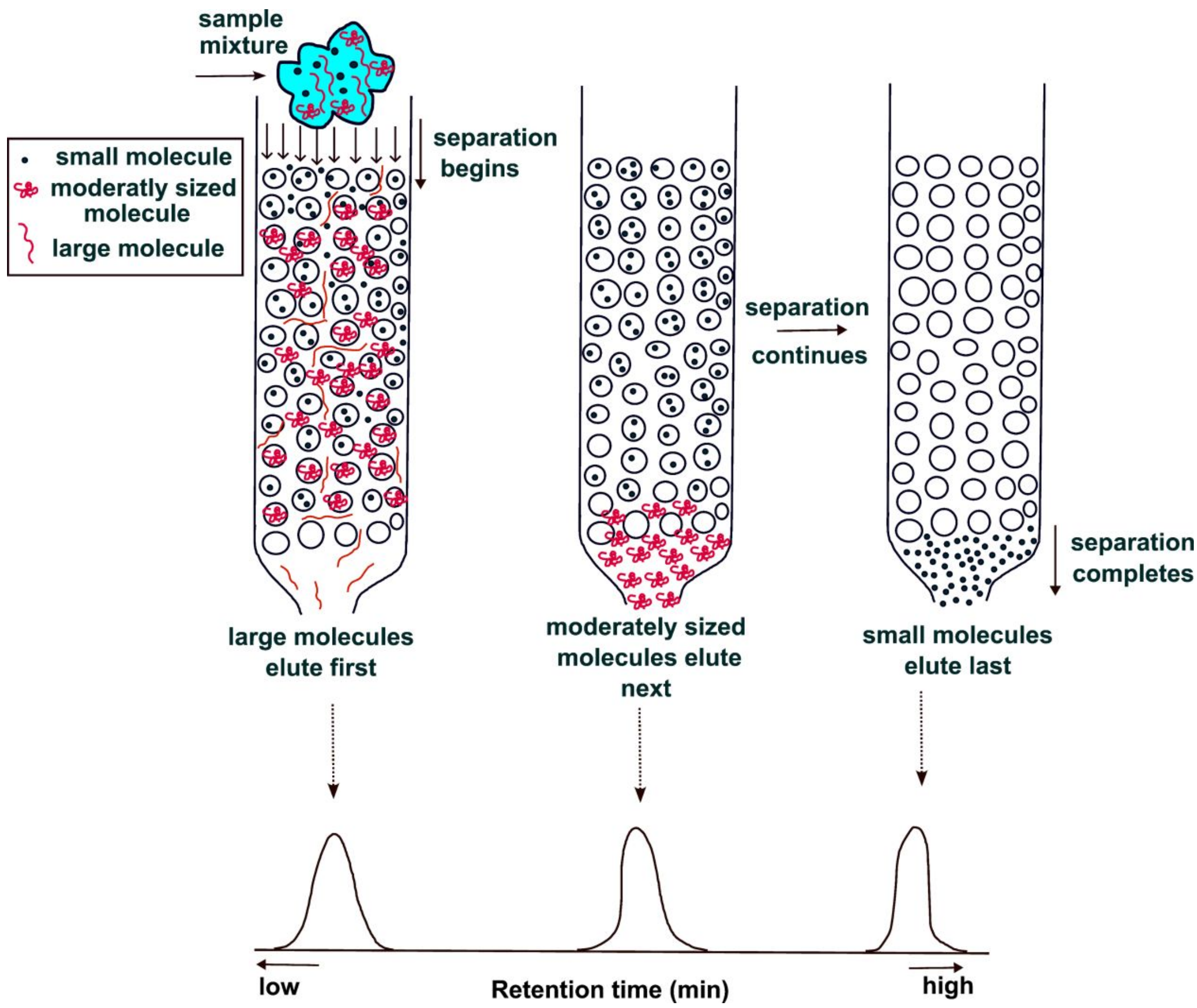

Figure S1. Schematic of the size exclusion or gel permeation chromatography (SEC/GPC) principle.

Discussed terminology:

Average Molecular Weights of Polymers.

Both natural and synthetic polymers consist of molecules that exhibit a distribution of molecular sizes rather than a single, uniform size. SEC typically provides two principal molecular weight averages: the number-average molecular weight (M_n) and the weight-average molecular weight (M_w). The value of M_n is defined as the total mass of the polymer sample divided by the total number of polymer molecules present. Mathematically, it is obtained by summing the product of the molecular weight of each polymer chain and the number of chains having that molecular weight, followed by division by the total number of chains, as shown in *equation S1*. Because M_n is averaged over the number of molecules, it is more sensitive to the presence of low-molecular-weight species.

$$M_n = \frac{\sum_{i=1}^{\infty}(N_i M_i)}{\sum_{i=1}^{\infty} N_i} \text{ ....} (equation\ S1)$$

Here, N_i = the number of polymer chains with a specific molecular weight, M_i

M_i = Molecular weight of a specific polymer chain

In contrast, M_W is calculated by summing the products of each chain's weight fraction and its molecular weight, or by summing the products of the number of chains times their molecular weight squared, all divided by the sum of the number of chains times their molecular weight, as shown in *equation S2*. Because M_w accounts for the square of molecular weight, it is more influenced by higher-molecular-weight polymers.

$$M_\omega = \frac{\sum_{i=1}^{\infty} N_i M_i^2}{\sum_{i=1}^{\infty} N_i M_i} \text{.........................}(equation\ S2)$$

The relative positions of M_n and M_w on a molecular weight distribution curve for a polymer are illustrated in **Figure S2**. The ratio M_w/M_n, known as dispersity (Đ), describes the breadth of the molecular weight distribution. A larger value of Đ indicates a broader distribution.

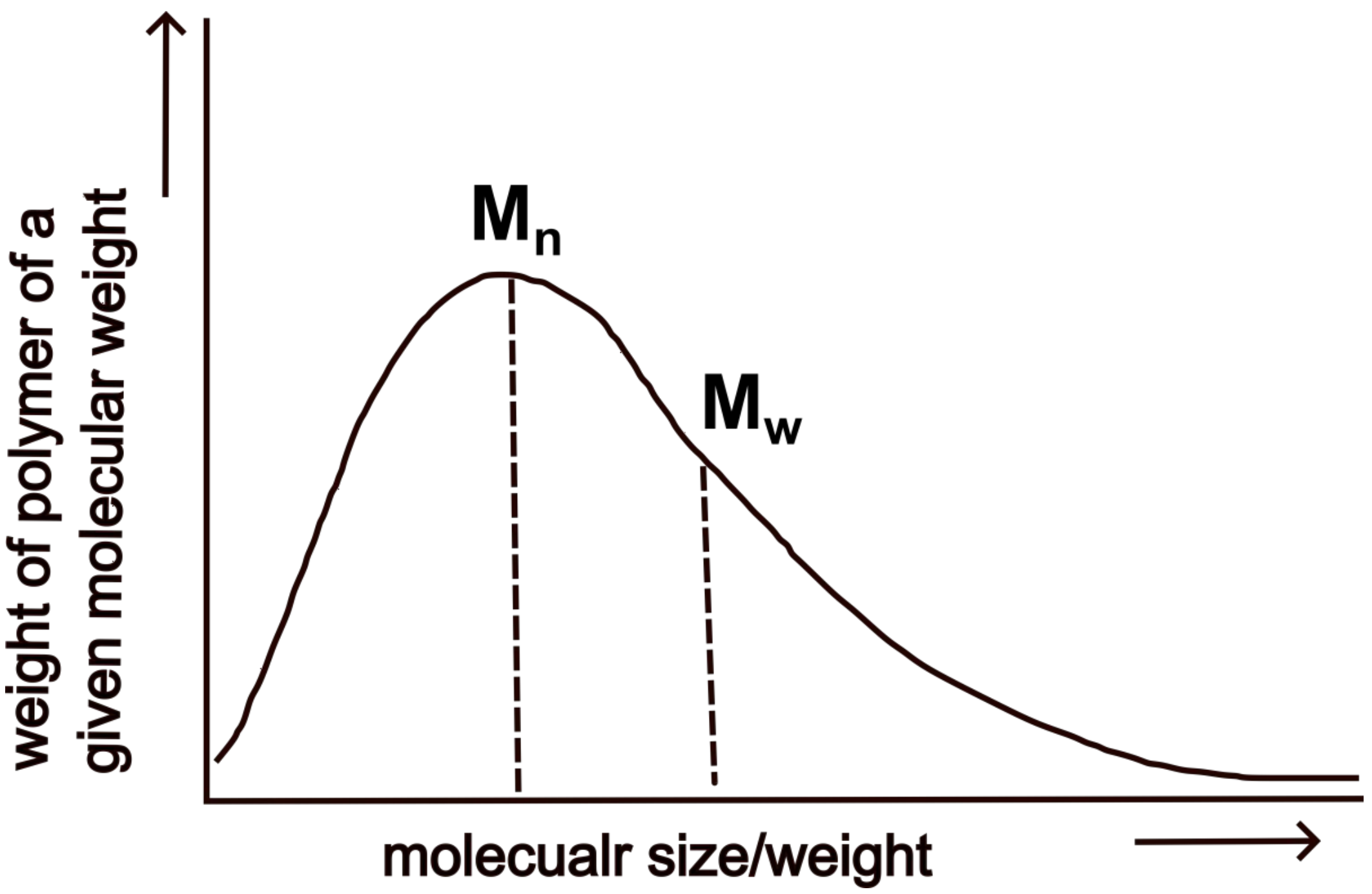

Figure S2. Relative positions of M_w and M_n on a molecular weight distribution plot.

Hydrodynamic Radius (R_h).
The hydrodynamic radius (R_h) describes the effective size of a polymer as it diffuses through a solvent. This value reflects not only the intrinsic dimensions of the polymer chain but its conformation, flexibility, and interactions with surrounding solvent molecules. In general, R_h increases with the molecular weight of the polymer. Additionally, compact or tightly coiled polymer conformations exhibit smaller R_h values compared to the extended or unfolded chains of similar molecular weight. Determination of R_h is valuable in the fields of biotechnology, drug delivery, and environmental science, where the solution behavior of macromolecules/polymers directly influences their functions and properties. Since SEC calculates molecular weights and intrinsic viscosities, R_h can be calculated using *equation S3*.

$$IV \cdot M = \frac{10\Pi N_A}{3} \times R_h^3 \text{.........................}(equation\ S3)$$

Here, IV = intrinsic viscosity *M = molecular weight* N_A *= Avogadro's number*

Detectors used in the Malvern OMNISEC Instrument
1. Refractive Index (RI) Detector: The refractive index (RI) detector is one of the most universally applicable detectors for SEC, as it enables the measurement of sample concentration by detecting changes in the refractive index (RI) of the mobile phase upon passage of the analyte. The RI signal is generated from the differential RI between the reference cell

(containing only the mobile phase solvent, THF in the present case) and the sample cell (containing the polystyrene mixture dissolved in THF), as illustrated in **Figure S3**. When the eluted sample enters the flow cell, light passing through the two compartments is refracted at slightly different angles; this refracted light is then reflected by a mirror onto positive and negative photodiodes in the detector, producing corresponding positive or negative peaks. The magnitude of the detector response is directly proportional to the sample concentration through the RI increment (*dn/dc*), as expressed in *equation S4*. The *dn/dc* value is characteristic of a specific polymer-solvent pair and is 0.185 mL g^{-1} for polystyrene (PS) in THF. Because this value is known, the concentration of the PS mixture can be quantified using the RI detector according to *equation S4*. Based on system and column calibration procedures performed with known standards, the RI detector subsequently provides relative molecular weight values for the analyte samples.

$$RI = \frac{k}{n_0} \cdot \frac{dn}{dc} \cdot C \ldots\ldots\ldots\ldots\ldots\ldots\ldots \text{(equation S4)}$$

Here, k= instrument constant *dn/dc = refractive index increment*

C = concentration (g/L) *n_0 = refractive index of solvent*

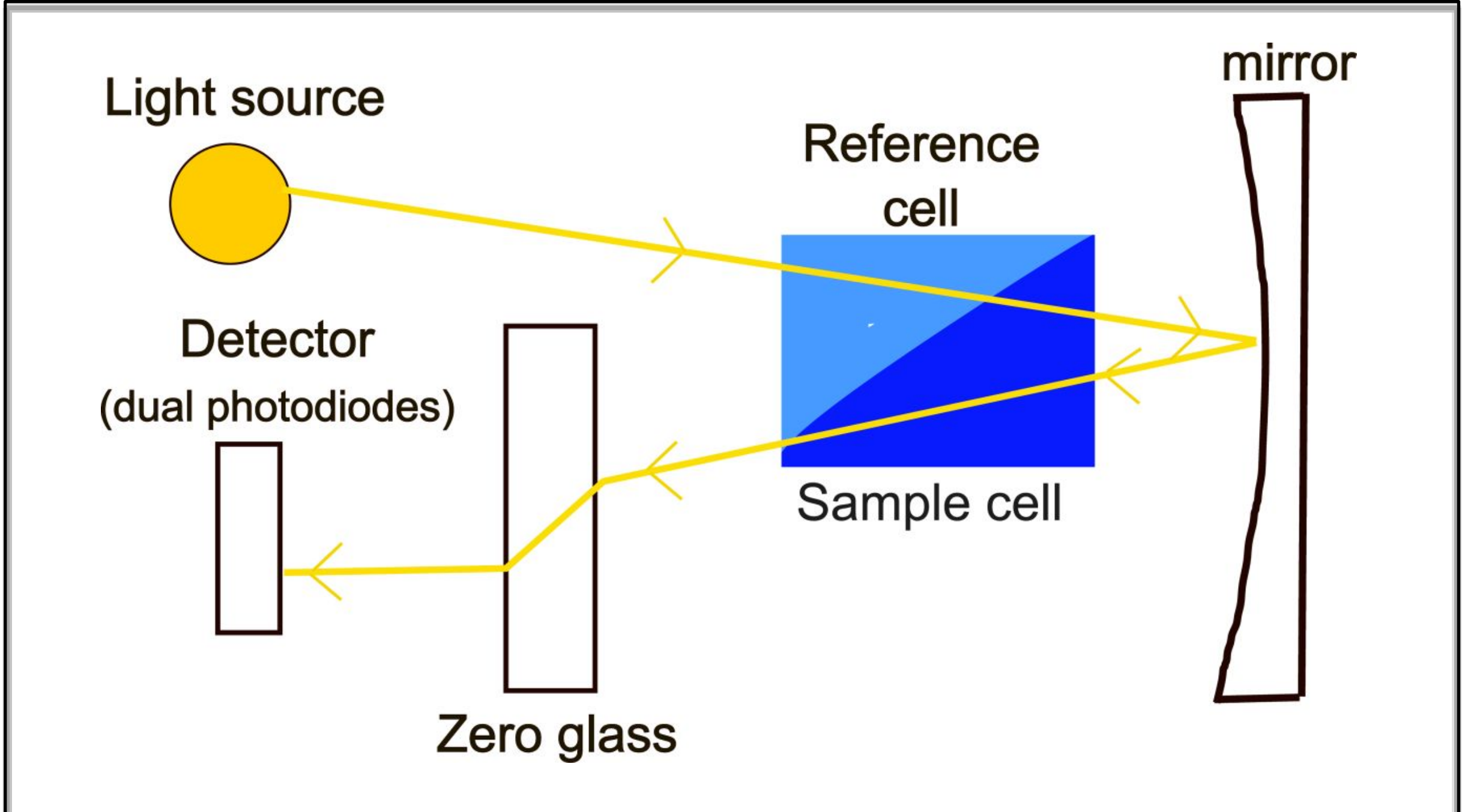

Figure S3. Refractive Index (RI) detector.

2. UV–Vis Photodiode Array (PDA) Detector: The UV–Vis photodiode array (PDA) detector is widely used for analytes, such as proteins or polymers that contain chromophores capable of absorbing light within the measurable wavelength range (190-900 nm), provided that the mobile phase does not significantly absorb in the same region. Using the Beer-Lambert law (*equation S5*), the PDA detector quantifies sample concentration based on the absorbance of the eluted species. In this context, the term dA/dcrepresents the absorption increment with respect to concentration, analogous to the refractive index increment (*dn/dc*) used in RI detection. This proportionality enables determination of sample concentration from the absorbance signal.

$$A = \frac{\varepsilon}{C}$$

$$\frac{dA}{dc} = \frac{\varepsilon}{Mw} \ldots\ldots\ldots\ldots\ldots\ldots\ldots\ldots \text{(equation S5)}$$

Here, A= absorbance *ε= molar extinction coefficient* *c = concentration*

3. Intrinsic Viscosity (IV) / Viscometer Detector: The viscometer detector measures changes in the viscosities of the eluting solutions and provides the intrinsic viscosity (IV) of the samples. IV reflects the contribution of the polymer to the solution's viscosity at infinite dilution and offers valuable insight into polymer conformation, chain flexibility, and the presence of branching. [4, 5] Because IV is inversely related to molecular density, viscometric data are particularly informative for distinguishing among polymer structures or shapes, such as compact, sphere-like coils versus more extended, fiber-like chains when comparing samples of similar molecular weight. Thus, the IV detector plays an important role in characterizing the size, shape, and conformation of polymer analytes in solutions.

4. Light Scattering Detector: The light scattering detector enables the determination of the absolute molecular weight of a polymer sample by measuring the intensity of light scattered by the eluting molecules, independent of polymer structure or the need for conventional calibration. In addition to the molecular weight determination, light scattering signals are also valuable for identifying aggregates or other high-molecular weight species present in the sample. Light scattering detectors can be configured at different scattering angles. A right-angle light scattering (RALS) detector measures scattered light at 90° and provides accurate molecular weight measurements for molecules with radii below approximately 10-15 nm. In contrast, a low-angle light scattering (LALS) detector collects scattered light at a much smaller angle (~7°), enabling accurate measurement of absolute molecular weights for all analytes without requiring angular extrapolation or fitting procedures.

Section S2: OMNISEC System Live Demonstration

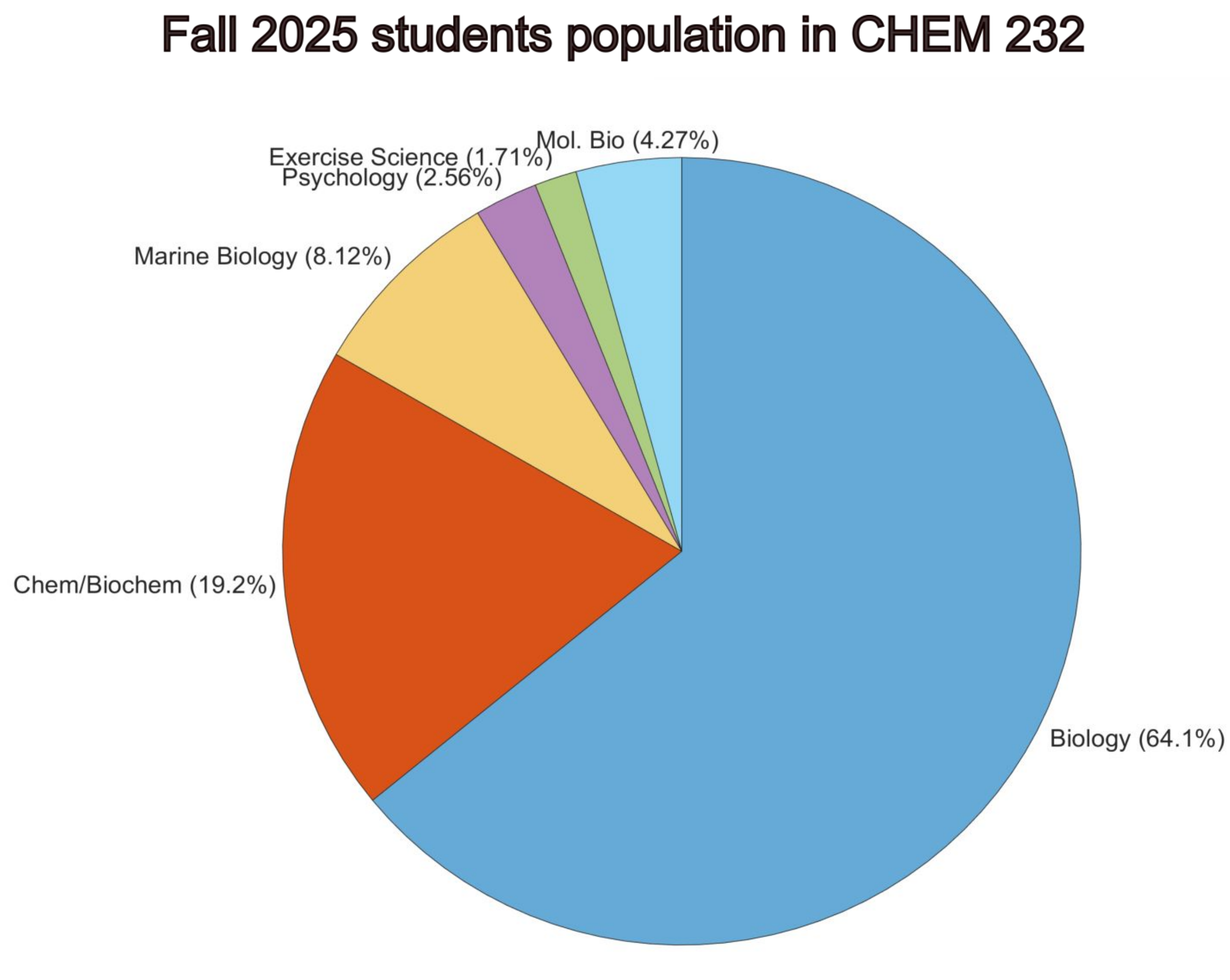

Figure S4. STEM major population (Total number of students enrolled 234) in the Experimental Organic Chemistry I Laboratory (CHEM 232) course in Fall 2025.

Table S1. Time requirements and personnel involvement for implementing the demonstration

Experimental steps	Time (hours)	Student involvement
**Sample preparation**: dissolution of polystyrene (PS) standards in THF at 40 ºC	~12 h (overnight dissolution)	No student attendance, completed in advance by 2 research assistants (RAs)
**Student Session 1:** Theoretical overview of SEC, and instrument/software sequence setup.	2 h	Participating students filtered pre-made PS solutions, transferred to autosampler vials, loaded, and completed OMNISEC sequence-entry procedure
**Instrument run/automated SEC sequence** for each session	~5 h per session	None (non-supervised sequence-controlled run, data later provided)
**Student Session 2:** Data analysis and interpretation	2 h	Students processed and interpreted chromatograms and completed the guided analysis/surveys/worksheets

Instrument Demonstration: Multidetector equipped OMNISEC instrument and its six core components are labeled as **1–6** in **Figure S5**. Students were introduced to its main two components: **(a)** the OMNISEC RESOLVE chromatography unit and **(b)** the OMNISEC REVEAL detector. The demonstration began with the RESOLVE system, which houses the pump, autosampler, and column. Students observed the degasser and isocratic pump (labeled as **1**), which ensures steady and stable mobile-phase flow, and the back-seal wash system that prevents salt precipitation. Tetrahydrofuran (THF) was used as the mobile phase (labeled as **2**) for the current demonstration. Waste containers and washing liquid bottles (filled with water: methanol mixture, 90:10 (v/v), labeled as **2**) were also shown to the students. Next, the autosampler (labeled as **3**), accommodating up to 96 vials at 4-60 °C, was explained along with its injection syringe, needle, sample loop, and insulated vial trays. Upon injection, students observed the sample passed into the T6000M mixed-bed GPC column (300 mm × 8 mm, 10 µm particle size, 10,000 Å pore size, 20,000,000 Da exclusion limit for polystyrene, labeled as **4**). For efficiency in the four-hour lab, only a single column was used to handle multiple samples and minimize solvent use. Students learned that while mesoporous silica is common in normal and reverse-phase HPLC, it is unsuitable here, as silica-based SEC columns are typically optimized for protein separation. The discussion highlighted that the T6000M column's porous styrene-divinylbenzene particles are compatible with a wide range of organic solvents and can operate at 30-60 °C without affecting pore size, particle morphology, or separation resolution, making it ideal for synthetic polymer analysis in educational and research settings. Next, students were introduced to the OMNISEC REVEAL detector (labeled **5**), which integrates four detectors including concentration, absorption, viscometer, and light scattering, that operate simultaneously for comprehensive sample characterization. The concentration of analytes can be measured via refractive index (RI) or UV absorption, while the light scattering detector provides absolute molecular weight. The viscometer determines IV, molecular density, and R_h, offering insights into molecular structure and conformation.

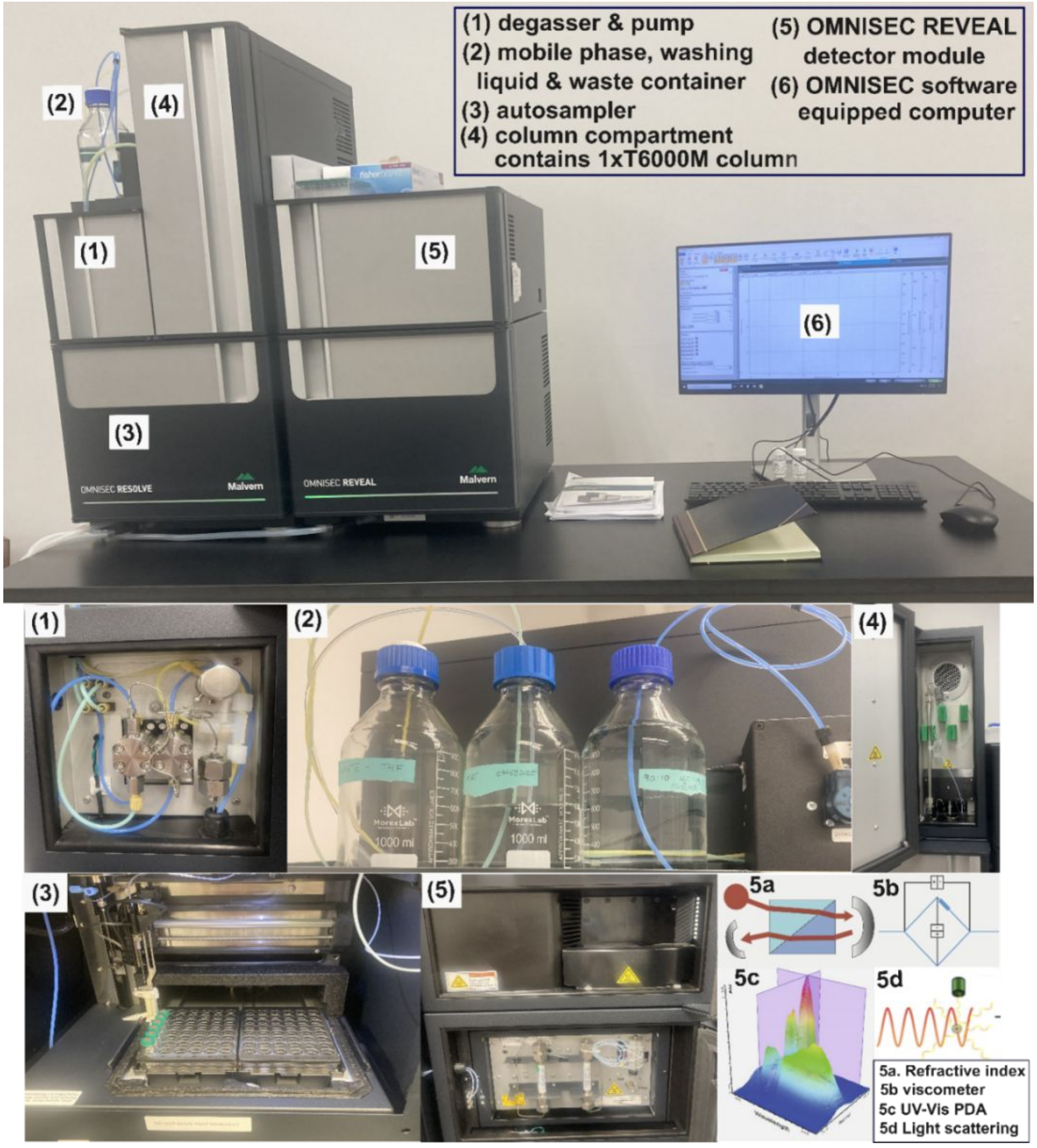

Figure S5. Photograph of the multidetector equipped MALVERN Panalytical OMNISEC system. Key components **(1–6)**, highlighted in the image and described in the legend, were introduced during the student demonstration. Images **5a-5d** were pulled from Malvern Panalytical website.

Section S3: OMNISEC Software Operation

Sample Enrollment and Sequence Start Instruction

Step 1: Navigate to the <u>Sequence</u> page and enter the required sample information. Specify the sample type, concentration, injection volume, number of injections, and the autosampler position. Example sample entry (collected in Fall 2025) is shown in **Figure S6**.

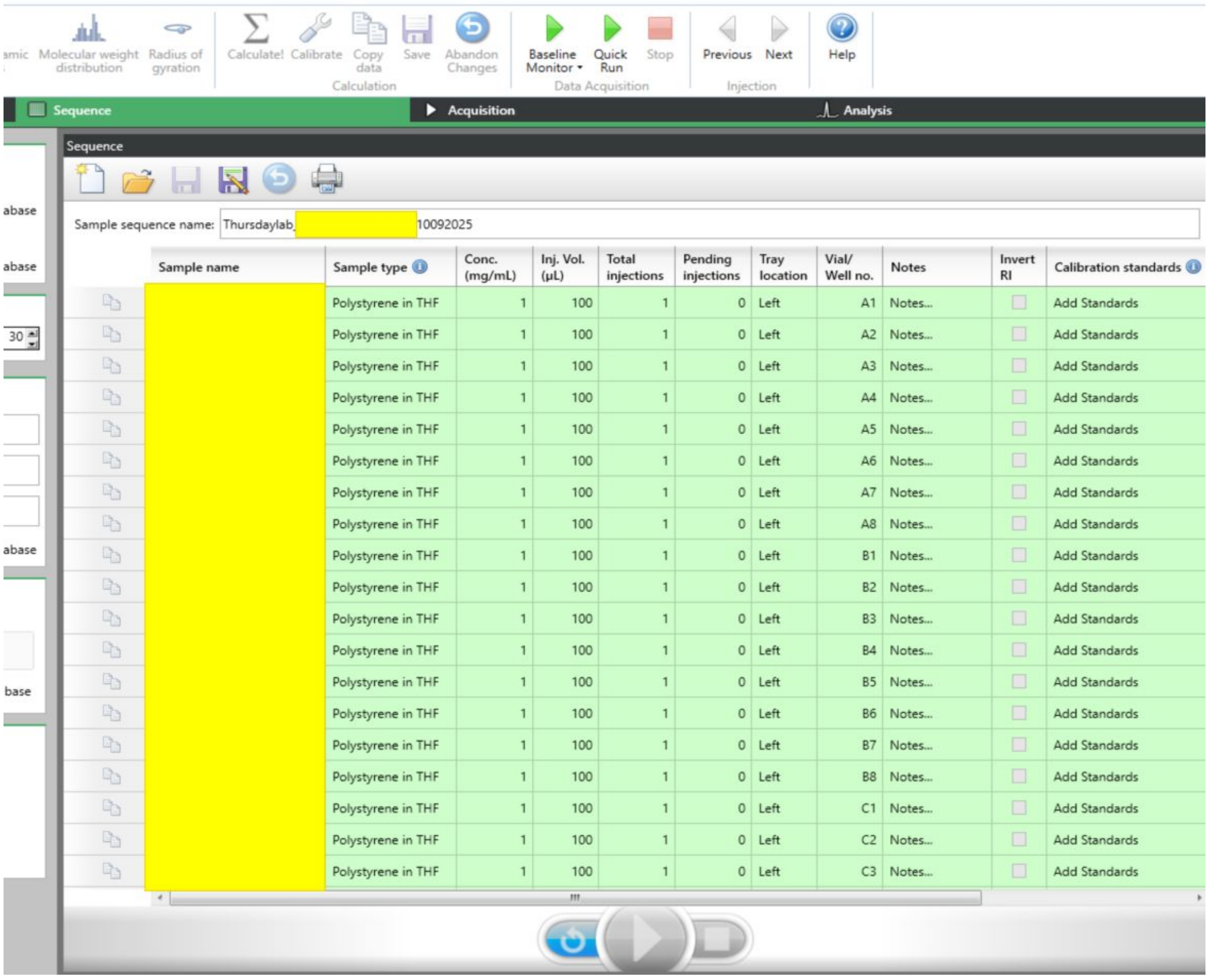

Figure S6. Sequence setup page.

Step 2: Select the appropriate run conditions. For the current demonstration, THF is used as the mobile phase with a refractive index (RI) of 1.405, a viscosity of 0.4023 mPa·s, and a flow rate of 1 mL min^{-1}. Choose the correct stationary phase column and set the total run time for 30 minutes. Adjust the temperatures of the autosampler, columns, and detectors as needed; in this example, students set temperatures of 23 °C for autosampler and 30 °C for columns and detectors. A representative run condition setup (collected in Fall 2025) is shown in **Figure S7.**

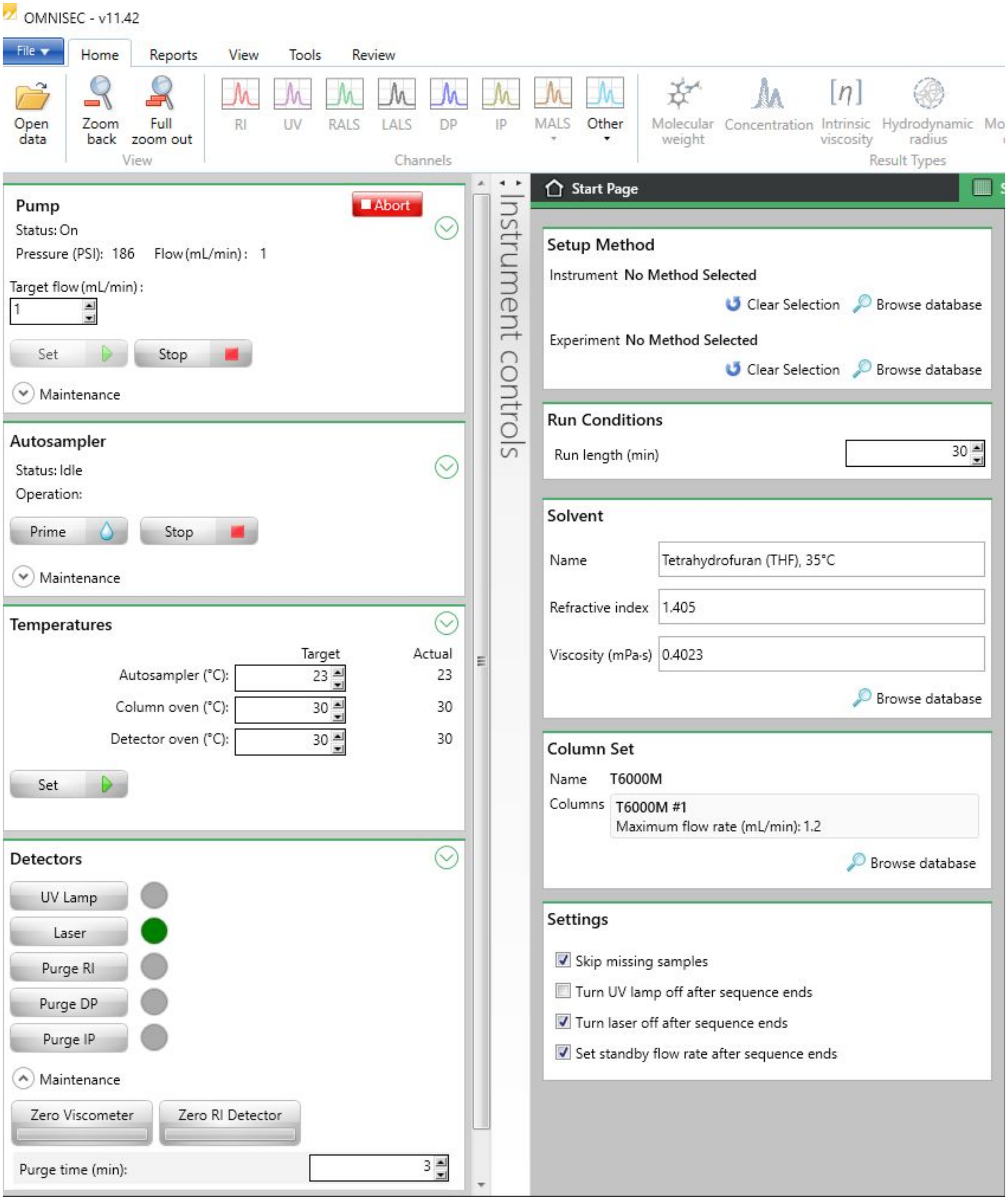

Figure S7. Run condition.

Step 3: Perform a 5-minute <u>baseline monitoring</u> of the detector signals. Before starting any measurements, verify that the baseline is stable and exhibits minimal noise. A representative baseline monitoring snapshot is shown in **Figure S8**.

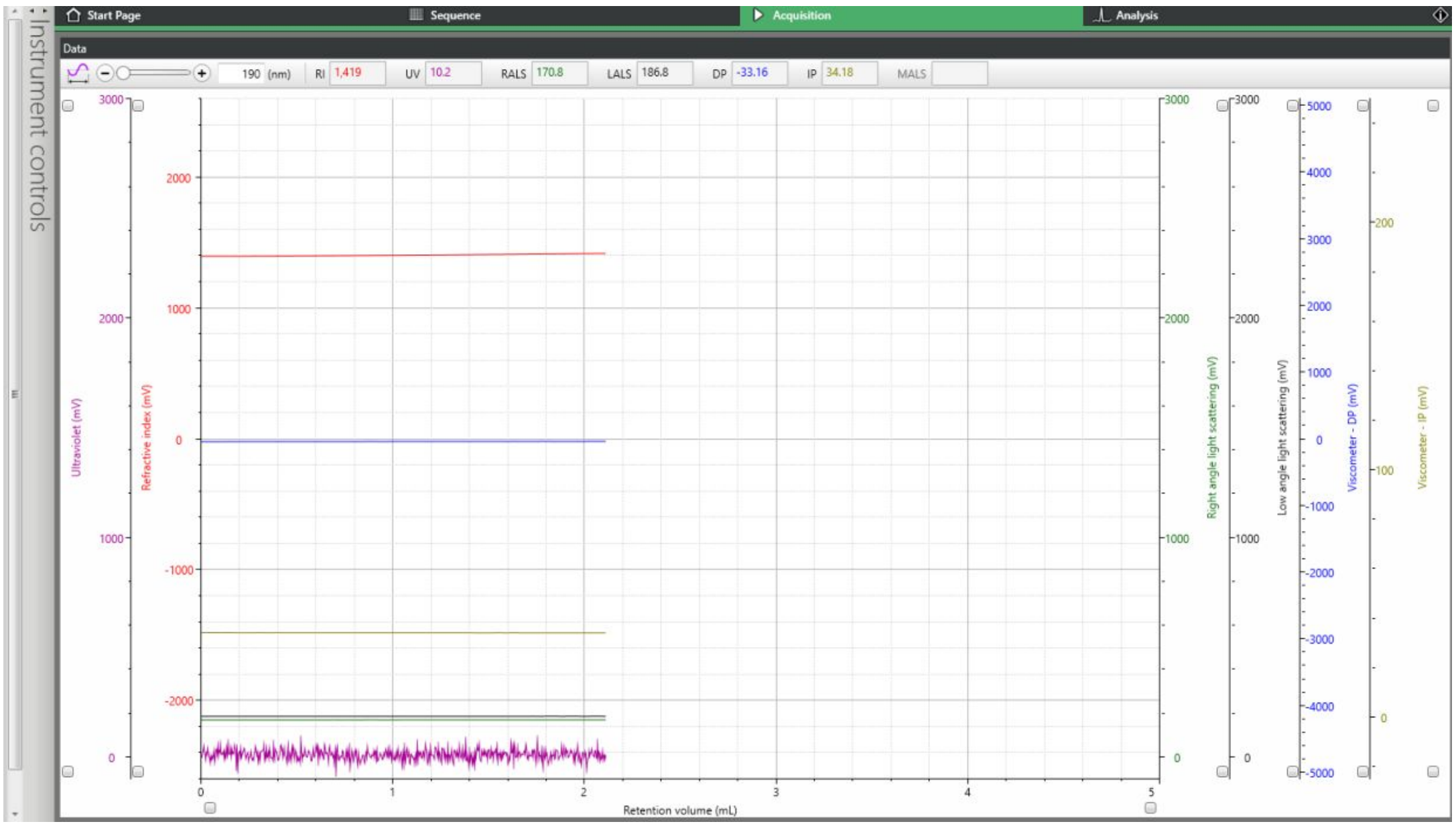

Figure S8. Baseline stability checks for detectors.

Step 4: Click the <u>Start</u> button (green) to begin the sequence (**Figure S9**). Once initiated, the system will automatically perform the following steps:

1. **Pre-injection wash:** The needle and whole injection pathway will be washed before the first injection.
2. **Between-sample wash:** The system will perform wash after each injection to prevent cross-contamination.
3. **Sample loading:** The sample loop will be filled with the specified injection volume.
4. **Data acquisition:** Data collection will begin automatically for each sample in the sequence.

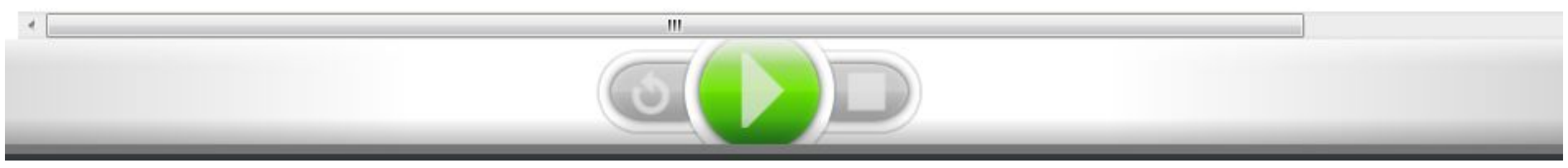

Figure S9. **Step 4**: Sequence starts.

Post-Measurement Data Acquisition

To retrieve and analyze data, follow the steps below.

Step 5: Navigate to the Analysis workspace and select Open Data to load your individual run sequence. Open both the sample file and a calibration standard file (**PS105k_rerun1_03312025**). The Analysis workspace consists of three windows: Raw Data, Derived Data, and Polymer Results; as shown in **Figure S10**. The Derived Data and Polymer Results sections will be empty at first as no analysis method has been created. Proceed to the next step to generate the method.

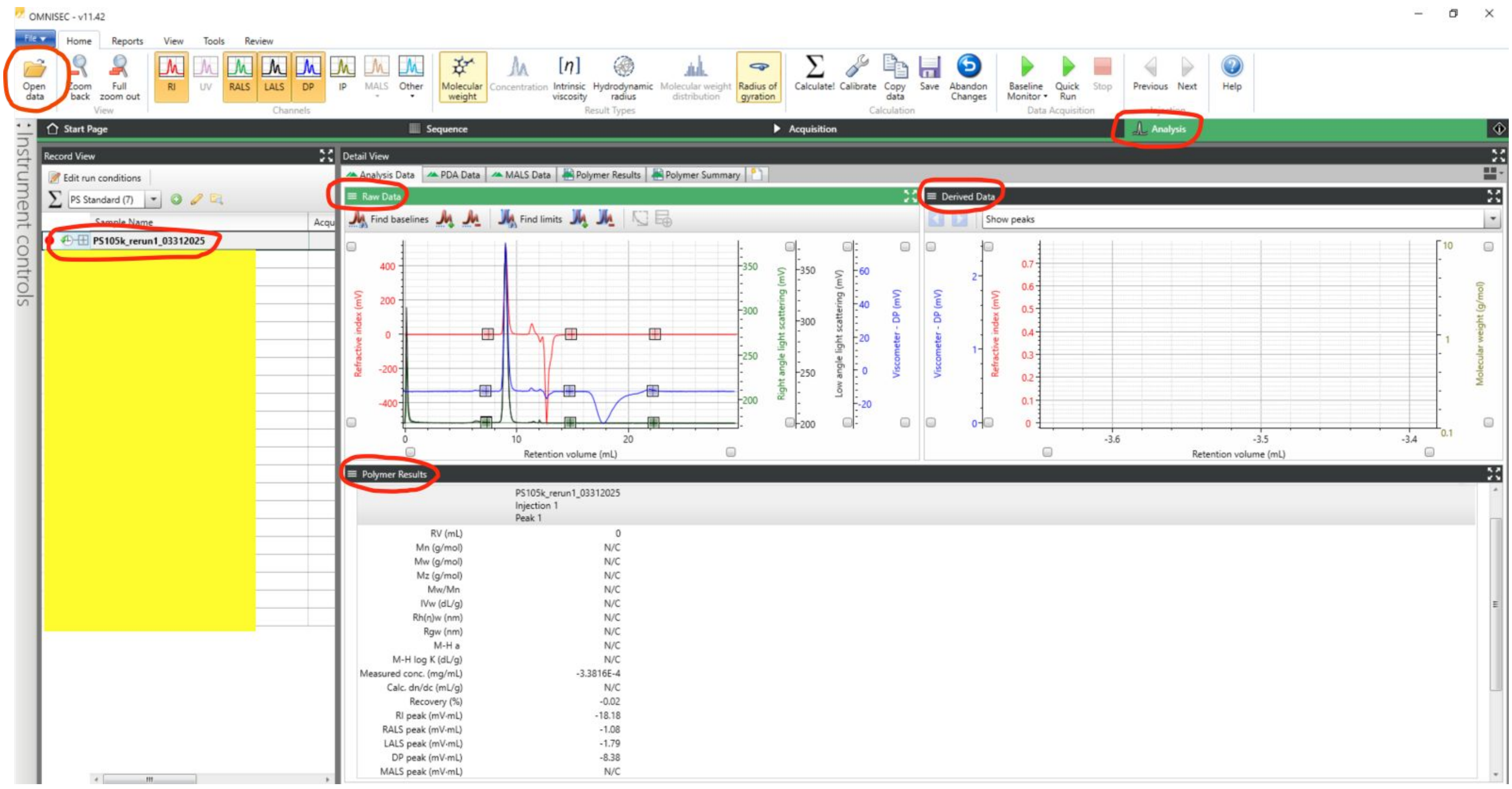

Figure S10. Data analysis steps are circled in red.

Step 6: Create a new Calculation Method by clicking the green + tab in most left windows shown in **Figure S11**. Assign an appropriate method name. Select the detectors RI, RALS, LALS, and DP (viscometer). Set the Calibration Type to Multidetector and choose Calculate concentration from sample *dn/dc* as the Analysis Type, since the analyte and its *dn/dc* value (polystyrene in THF) are known. In this step, detector calibration, including offset adjustment, response factor determination, and band-broadening corrections are performed simultaneously. Band-broadening correction in SEC chromatogram is calculated and applied only for multi-detector analyses, such as, universal calibration and multi-detection with light scattering. This correction accounts for the convolution of the sample's true molecular size distribution and system-induced dispersion effects (e.g., column band broadening, mass transfer limitations, etc.). Without this correction, such effects can artificially distort peak width and shape, leading to inaccurate calculation of molecular weights, and dispersity.

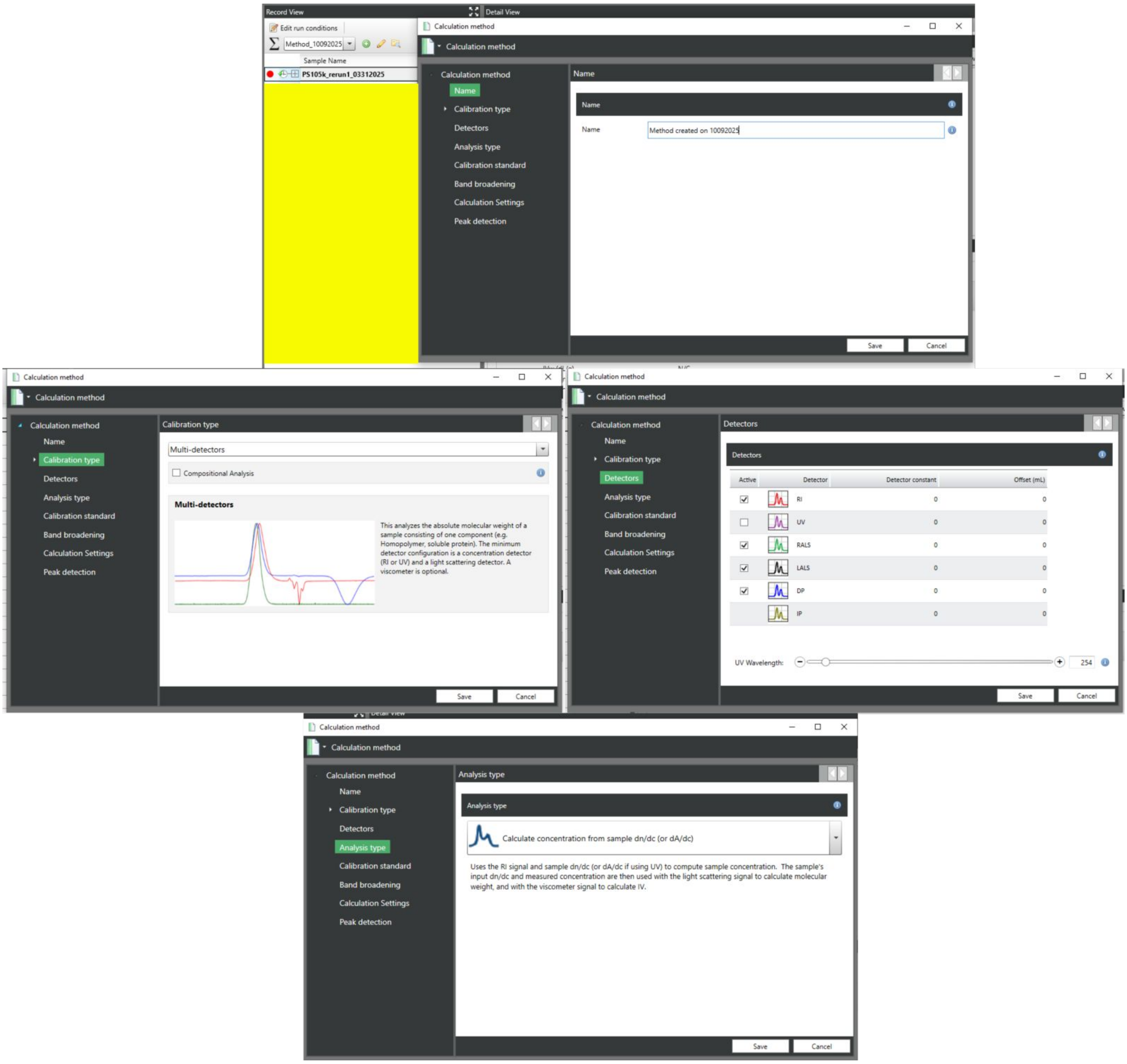

Figure S11. Method creation steps.

Step 7: Load the created calculation method. Select the **PS105k_rerun1_03312025** calibration standard data and click Find limits (circled in red). Software will set the baseline limits for the chromatogram. The calculated results will now appear in the Derived Data section.

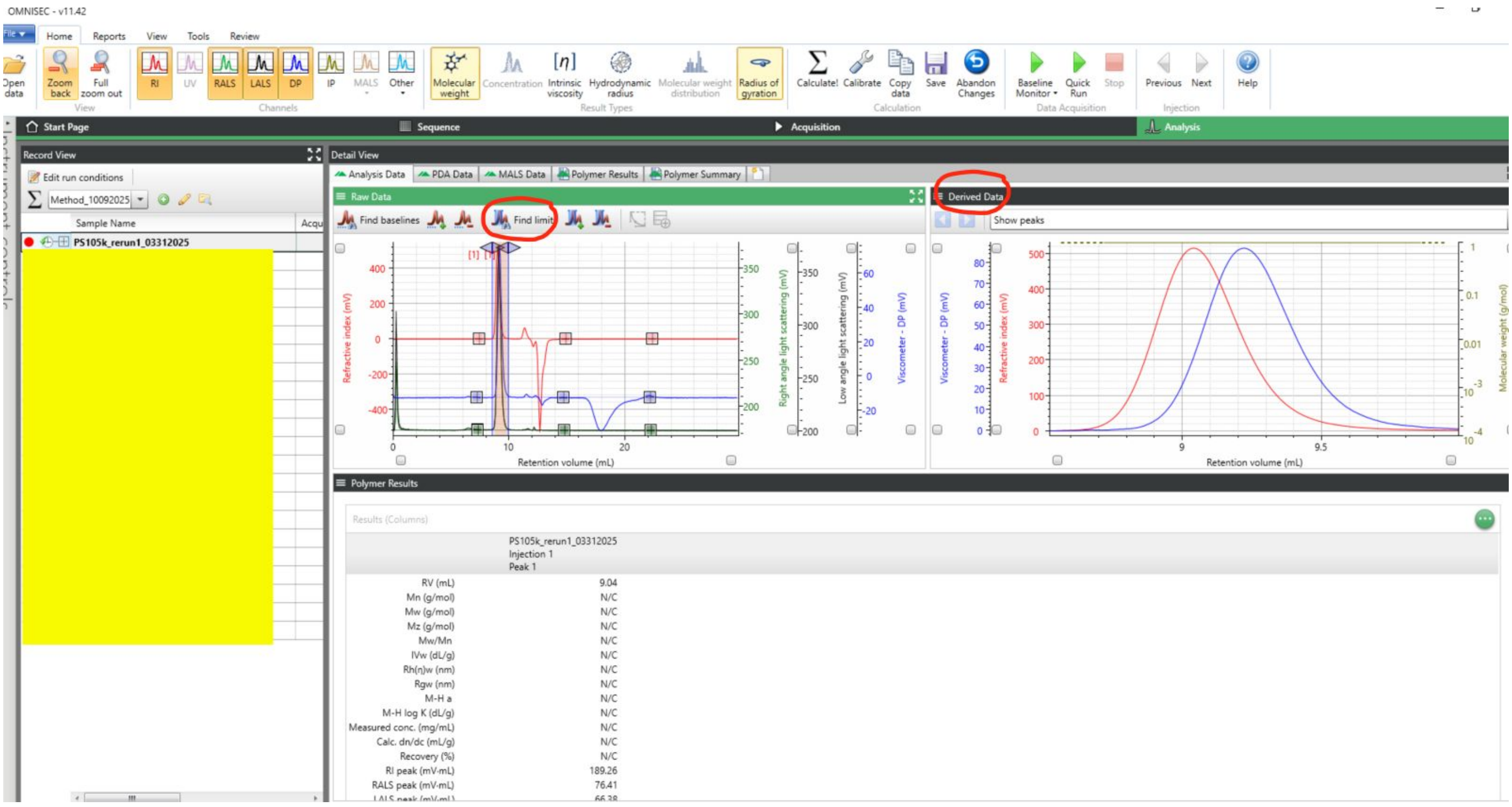

Figure S12. Find limit on the **PS105k_rerun1_03312025** standard data.

Step 8: Click Calibrate, then select the calibration standard **Polystyrene, 105 kDa, in THF, 30 °C** from the database. Confirm your selection to apply the calibration.

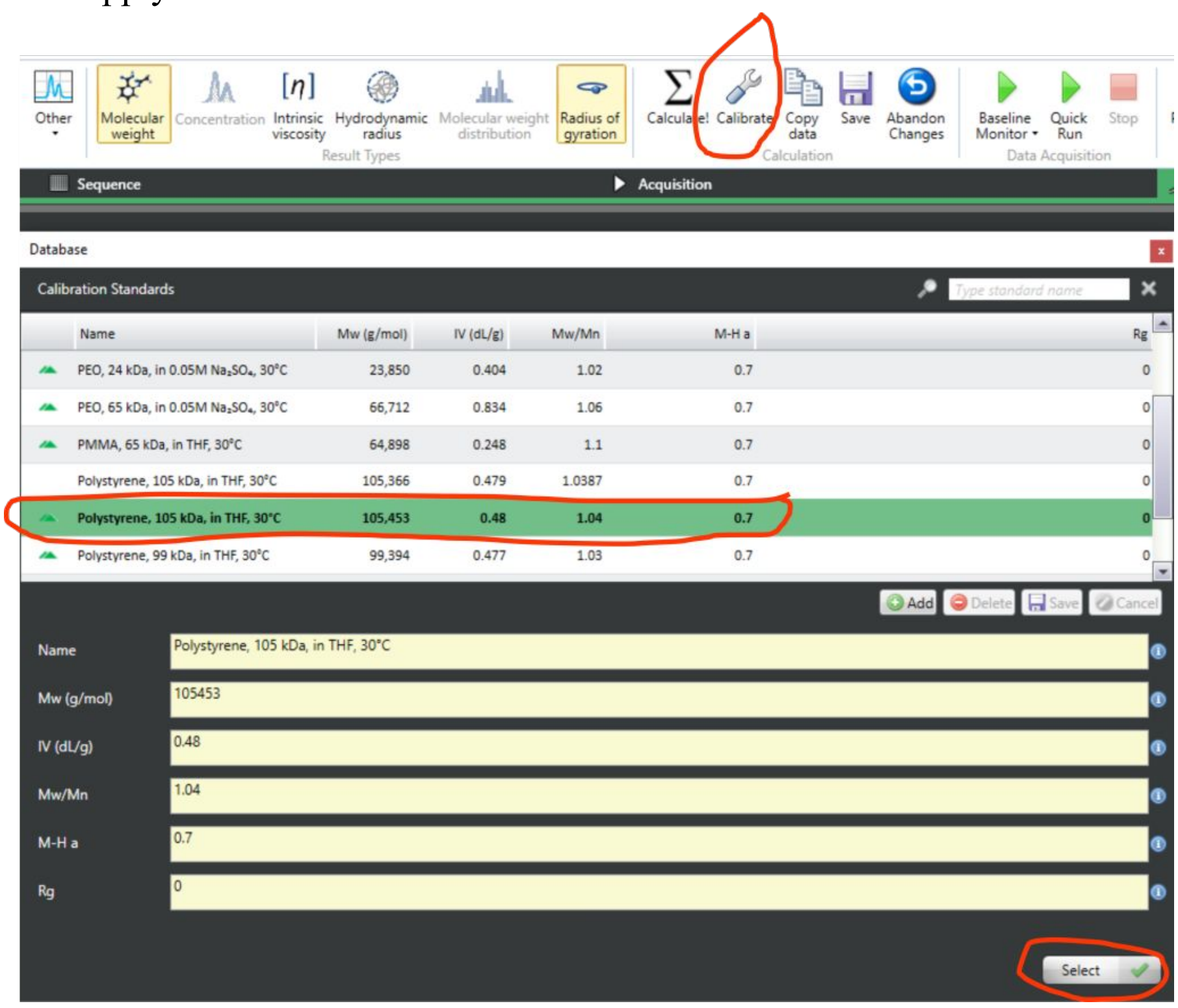

Figure S13. Selection of calibration standards from the database.

Step 9: After confirming the calibration standard, software will perform the method calibration. The updated results will appear in the **Polymer Results** windows. A representative result from a Fall 2025 session is shown as, M_n = 101.7k g mol^{-1}, M_w = 105.8k g mol^{-1}, Đ = 1.041 and IV = 0.478 dL g^{-1} (measured at 30 ºC) whereas the vendor-provided standard details are M_n = 101.2k g mol^{-1}, M_w = 104.7k g mol^{-1}, Đ = 1.036 and IV= 0.475 dL g^{-1} (measured at 40 ºC). The measured values for the **PS105k_rerun1_03312025** validate the accuracy of the created method and calibration settings.

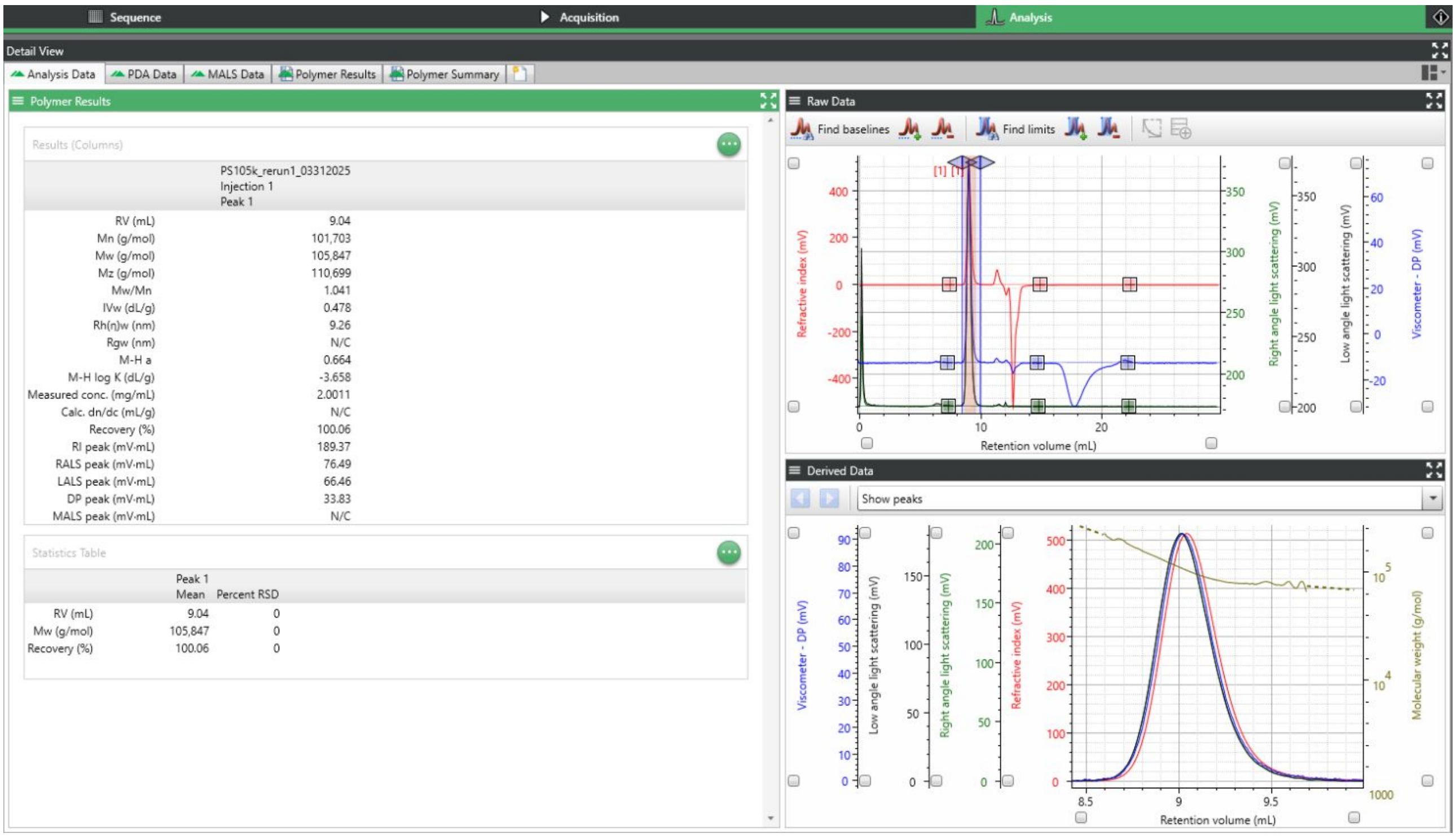

Figure S14. Results for **PS105k_rerun1_03312025** confirm the validity of the calibration method.

Step 10: After verifying the created method and the calibration standard results accurately, proceed to analyze the unknown sample. Select your sample in the Record View and manually set the baseline limits around the peaks. Record or copy the Polymer Results and the corresponding chromatogram into your worksheet. For viewing the Derived Data, select the RI detector only to obtain a clean, non-overlapping signal that is easier to interpret.

Section S4
Representative Data Generated by Participating Students in Fall 2025

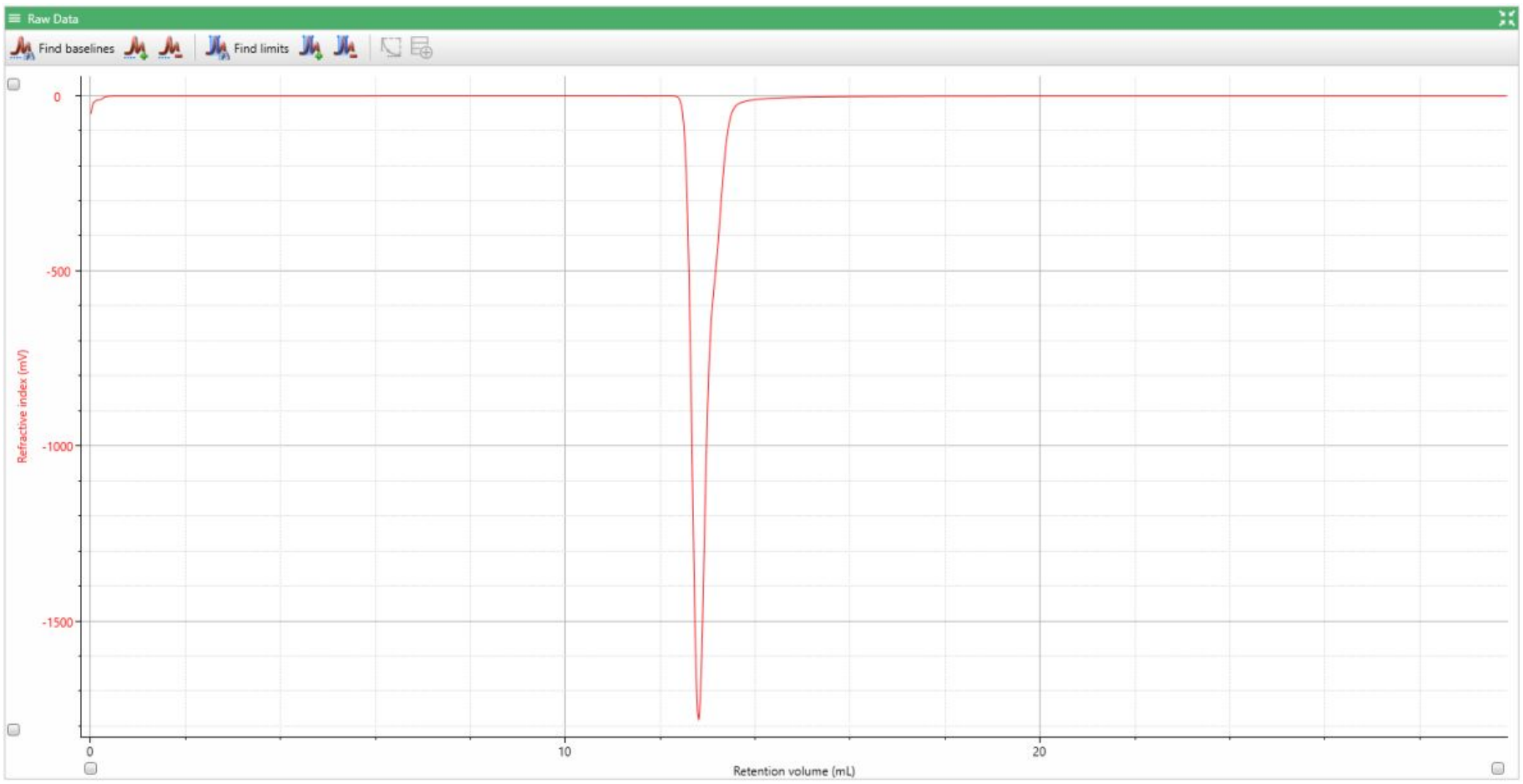

Figure S15. A representative student-generated data showing no RI chromatogram for PS mixture injection.

Table S2. Triple detector and universal calibration analyzed results of the PS mixture.

Detail View

Analysis Data | PDA Data | MALS Data | Polymer Results | Polymer Summary

Polymer Results

Results (Columns)

	Injection 1<br>Peak 1	Peak 2	Peak 3
RV (mL)	6.61	8.73	10.14
Mn (g/mol)	4,531,794	217,334	11,814
Mw (g/mol)	4,745,479	244,008	13,006
Mz (g/mol)	4,937,602	273,994	13,610
Mw/Mn	1.047	1.123	1.101
IVw (dL/g)	7.4016	0.7649	0.047
Rh(η)w (nm)	81.94	14.16	2.32

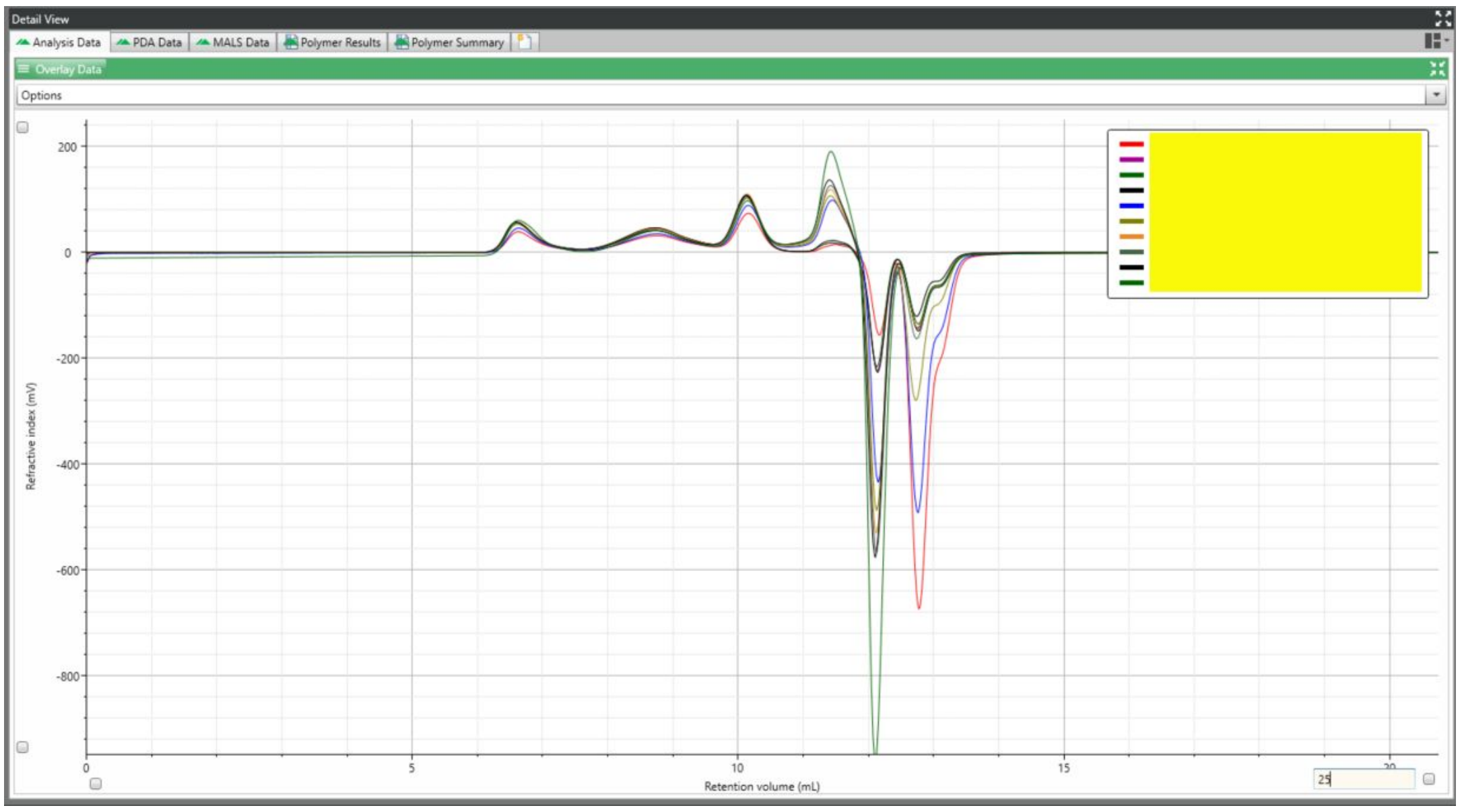

Figure S16. An overlay comparison of randomly picked 10 students teams generated SEC data (obtained using RI detector and conventional calibration method) during the demonstration in Fall 2025, along with their corresponding statistical results (**Table S3**) evaluate the degree of agreement between their individual measurements and the overall high precision of the dataset.

Table S3. Generated datasheet from the overlay graph, shown in **Figure S16**. The Result Statistics Table presents the average retention volumes (RV), M_w and % recovery along with their % relative standard deviations (RSD) of all 10 injections.

Polymer Results

Results (Columns)

	Injection 1 Peak 1	Peak 2	Peak 3	Injection 1 Peak 1	Peak 2	Peak 3	Injection 1 Peak 1	Peak 2	Peak 3
RV (mL)	6.64	8.75	10.16	6.61	8.73	10.14	6.6	8.72	10.13
Mn (g/mol)	4,180,580	140,814	10,091	4,294,095	143,673	10,529	4,172,330	144,694	10,598
Mw (g/mol)	4,638,286	243,893	11,990	4,775,247	265,016	12,599	4,792,964	266,988	12,908
Mz (g/mol)	5,060,841	400,397	14,215	5,216,829	470,154	14,955	5,366,383	462,261	15,535
Mw/Mn	1.109	1.732	1.188	1.112	1.845	1.197	1.149	1.845	1.218

Polymer Results

Results (Columns)

Injection 1 Peak 1	Peak 2	Peak 3	Injection 1 Peak 1	Peak 2	Peak 3	Injection 1 Peak 1	Peak 2	Peak 3	Injection 1 Peak 1	Peak 2	Peak 3	Injection 1 Peak 1	Peak 2	Peak 3
6.62	8.73	10.14	6.64	8.76	10.16	6.62	8.73	10.14	6.62	8.73	10.14	6.62	8.74	10.14
4,242,722	158,431	10,630	4,517,093	151,570	9,946	4,189,503	139,378	10,281	4,160,544	138,094	10,231	4,239,948	141,420	9,867
4,737,290	267,901	13,400	4,837,693	226,223	11,485	4,694,386	256,591	12,356	4,687,291	246,333	12,161	4,718,190	266,337	11,886
5,187,324	447,195	17,159	5,160,245	321,758	13,139	5,150,149	444,727	14,762	5,155,451	405,168	14,265	5,154,198	483,652	14,033
1.117	1.691	1.261	1.071	1.493	1.155	1.121	1.841	1.202	1.127	1.784	1.189	1.113	1.883	1.205

Injection 1 Peak 1	Peak 2	Peak 3	Injection 1 Peak 1	Peak 2	Peak 3
6.61	8.72	10.13	6.64	8.74	10.15
4,274,887	140,626	10,638	4,081,241	134,797	9,820
4,786,581	268,937	12,553	4,481,908	230,207	11,720
5,244,762	486,568	14,692	4,805,479	377,570	13,785
1.12	1.912	1.18	1.098	1.708	1.193

Statistics Table

	Peak 1 Mean	Percent RSD	Peak 2 Mean	Percent RSD	Peak 3 Mean	Percent RSD
RV (mL)	6.62	0.1931	8.74	0.1367	10.14	0.1208
Mw (g/mol)	4,714,984	2.142	253,843	6.384	12,306	4.708

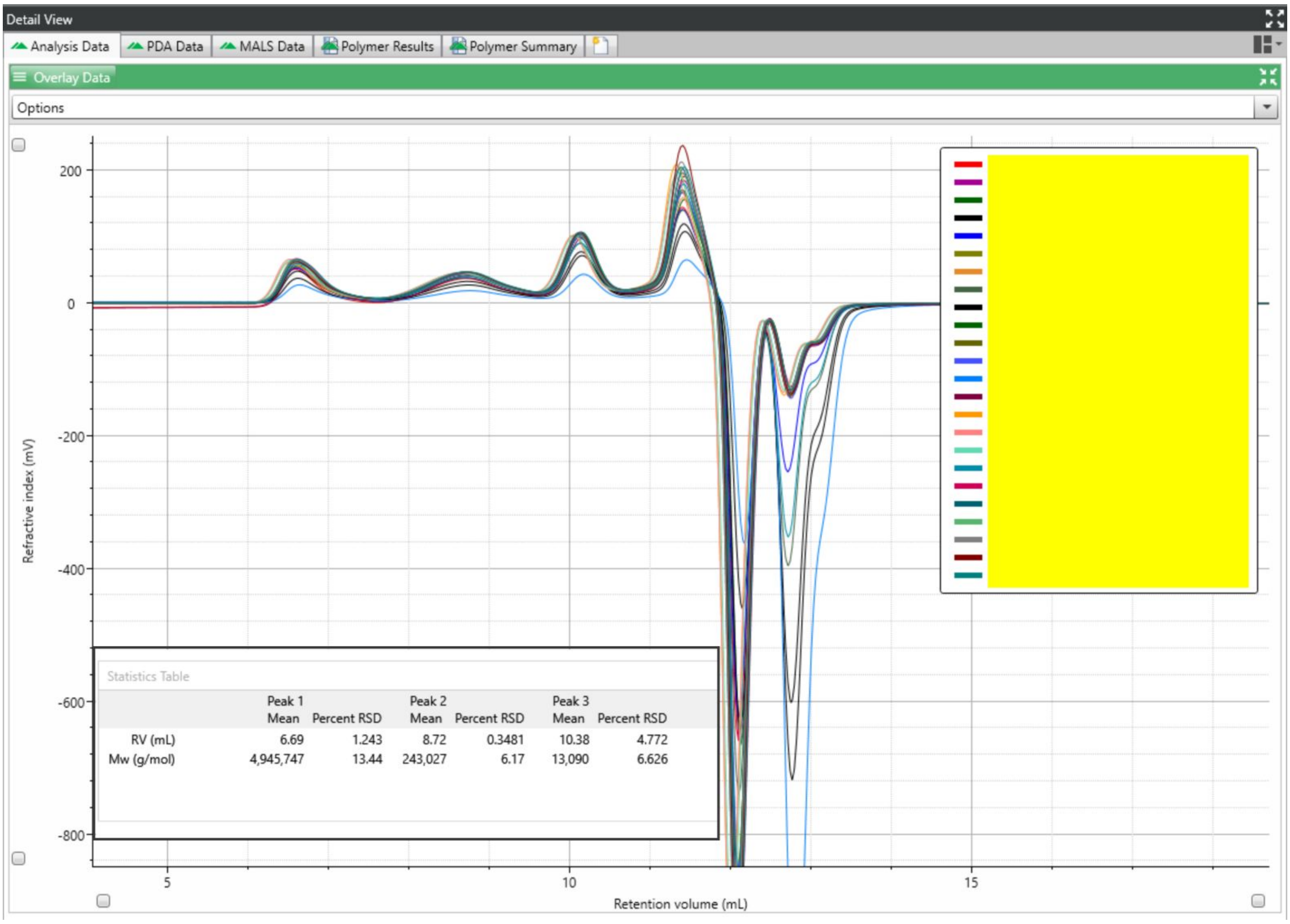

Figure S17. Comparison of 24 SEC datasets obtained using the RI detector and conventional calibration method during Summer 2026 demonstration. First 10 datasets represent measurements collected by representative 10 student teams, whereas the other 14 datasets were acquired independently by the instructor for comparison and validation purposes.

Section S5: Post-Demonstration Worksheets

Post-demonstration worksheet Distributed during Fall 2025 demonstration

Size Exclusion Chromatography Demonstration
Post lab Questions

1. You are given a mixture containing three polystyrene (PS) standards with $M_{w,PS1}$ = 65,500g mol^{-1}, $M_{w,PS2}$ = 17,800 gmol^{-1} and $M_{w,PS3}$ =200,000 gmol^{-1}. How would you separate them based on the size exclusion principle. Assuming you are provided with a column having an exclusion limit of 600,000 gmol^{-1}, how does the resulting chromatogram appear? Draw a chromatogram illustrating the expected elution order based on size exclusion principle.

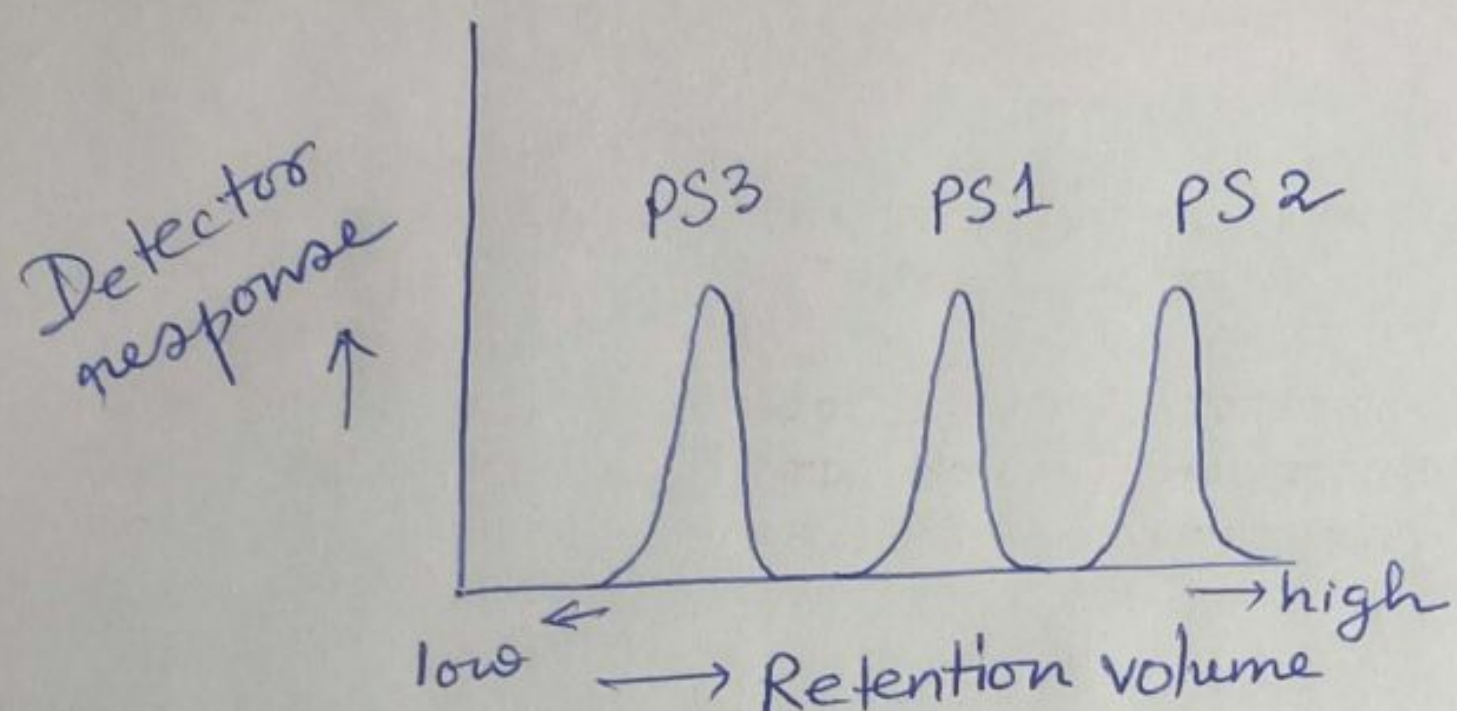

2. If you are provided with a new column that has an exclusion limit of 100,000 gmol^{-1}, Which polymer standard in the above-mentioned mixture would be excluded from the column?

PS 3

3. You have synthesized a polystyrene sample in the lab and would like to determine its molar mass distribution (Đ). After running the sample in OMNISEC instrument and characterizing it using the RI detector, you obtain molar masses of that sample; M_n = 15,500 g mol^{-1} and M_w = 18,000 gmol^{-1}. What is the calculated Đ of the sample, and how would you describe the expected appearance of the polystyrene elution peak based on this value?

$$Đ = M_w/M_n = \frac{18000 \text{ gmol}^{-1}}{15,500 \text{ gmol}^{-1}} = 1.16$$

Narrow & monomodal peak

Pre-demonstration worksheet (answers are highlighted)

Size Exclusion Chromatography (Demonstration)
Pre-Demonstration Worksheet, Summer 2026

Choose 1 answer only **Name:**

1) What is the main separation mechanism in Size Exclusion Chromatography (SEC)?
A. Charge differences B. Polarity differences
C. Molecular size differences D. Melting point differences

2) In SEC, larger molecules generally:
A. elute first B. elute last
C. Do not elute D. Decompose in the column

3) Why do larger molecules elute/extract earlier in SEC?
A. They interact strongly with the stationary phase B. They enter more pores of the stationary phase
C. They enter fewer pores and travel a shorter path D. They have lower molecular weights

4) A chromatogram shows three peaks with the following elution volumes:
Peak A: 45 mL **Peak B: 65 mL** **Peak C: 90 mL**
Which peak most likely corresponds to the smallest molecule.
A. Peak A B. Peak B C. Peak C

5) What does M_n represent in polymer or macromolecular science?
A. Maximum molecular weight B. Number-average molecular weight
C. Minimum molecular weight D. Molecular number

6) Dispersity (Đ) is related to:
A. Polymer color B. Polymer solubility
C. Breadth of molecular weight distribution D. Polymer density

7) Which factor is least likely to affect SEC separation?
A. Column dimensions B. Pore size of the stationary phase
C. Sample injection volume D. Color of the sample

8) The molecular weight of proteins is commonly expressed in:
A. Volts B. Kilodaltons (kDa)
C. Moles per liter D. Degrees Celsius

9) Which protein would likely separate first?
A. Insulin (~6 kDa) B. Lysozyme (~14 kDa) C. Bovine serum albumin (~66 kDa)

10) A polymer sample containing chains of many different lengths is:
A. Monodisperse B. Polydisperse C. Cross-linked

Post-demonstration worksheet (answers are highlighted)

Size Exclusion Chromatography (Demonstration)
Post-Demonstration Worksheet Summer 2026

Choose 1 answer only **Name:**

1) A mixture contains: Protein A: 150 kDa; Protein B: 70 kDa; Protein C: 15 kDa. What is the expected elution order in OMNISEC analyzed chromatogram?

A. C → B → A B. B → A → C C. A → B → C

2) OMNISEC datasheet revealed the data of a polymer sample as: M_n = 50,000 g/mol and M_w = 100,000 g/mol. What is your predicted dispersity for this sample?

A. 0.5 B. 2.0 C. 50,000 D. 150,000

3) What happens if you accidentally inject too much sample in OMNISEC instrument?

A. Resolution improves B. Peaks become broader and may overlap
C. Elution volume decreases dramatically D. Molecular weight increases

4) Which detector is used to determine intrinsic viscosity in the OMNISEC system?

A. Viscometer B. UV detector C. RI detector

5) A large protein is unable to enter most of the pores within an SEC column packing material. What effect would this have on its elution volume?

A. It would elute later because it interacts strongly with the packing material
B. It would elute earlier because it follows a shorter path through the column
C. It would remain trapped inside the column D. It would decompose before detection

6) Which detector in OMNISEC provides information about the absolute molecular weight of a protein?

A. UV B. RI C. Multi-angle light scattering D. Fluorescence

7) A mixture contains bovine serum albumin (66 kDa), lysozyme (14 kDa), and insulin (6 kDa). Which protein would be expected to elute last from an SEC column?

A. Insulin B. Lysozyme C. Bovine serum albumin

8) Two polymer samples have identical M_n values. Sample A has Đ = 1.05 and Sample B has Đ = 2.8. Which statement is most accurate?

A. Sample A contains a broader molecular weight distribution.
B. Sample B contains a broader molecular weight distribution.
C. Both samples have identical molecular weight distributions.
D. Dispersity does not provide information about molecular weight distribution.

9) What information does the RI detector primarily provide?

A. Sample concentration B. Sample color C. pH D. Conductivity

10) Why is the mobile phase filtered and degassed before use in OMNISEC?

A. To change the molecular weight B. To prevent bubbles and contamination
C. To increase sample concentration D. To change the detector wavelength

Section S6: Instructor's Notes

Conventional Calibration

A series of well-characterized polystyrene standards with known molecular weights were analyzed using the RI detector. These narrow and broad standards **(Table S4)** span a wide range of molecular sizes and masses to encompass the full resolution window of the column. Resulting data were used to construct a conventional calibration curve for subsequent sample analysis. Here chromatograms are anchored from the injection starting point (0 mL), and because the solvent peaks consistently appeared at ~12 and ~12.75 mL with highly linear calibration curves (R2 = 0.9969) (**Figure S18**), it is confidently concluded that the flow rate was sufficiently stable and that the absence of a small molecular weight standards or flow markers (such as Toluene, BHT) does not compromise the reliability of the data.

Table S4. PS calibration standards.

**Narrow & Broad Standards**	**M_p (gmol^{-1})**	**M_n (gmol^{-1})**	**M_w (gmol^{-1})**	**Đ**
PS1200	1,200	1,160	1,240	1.069
PS2400	2,400	2,360	2,000	0.847
PS5600	5,640	5,510	5,780	1.049
PS13k	12,630	12,260	13,000	1.06
PS29k	29,100	28,300	30,000	1.06
PS105k	---	101,166	104,716	1.035
PS245k	---	104,782	245,239	2.34
PS389k	388,500	377,400	400,000	1.059
PS874k	874,200	849,000	900,000	1.06
PS1880k	1877,800	1790,000	1970,000	1.10

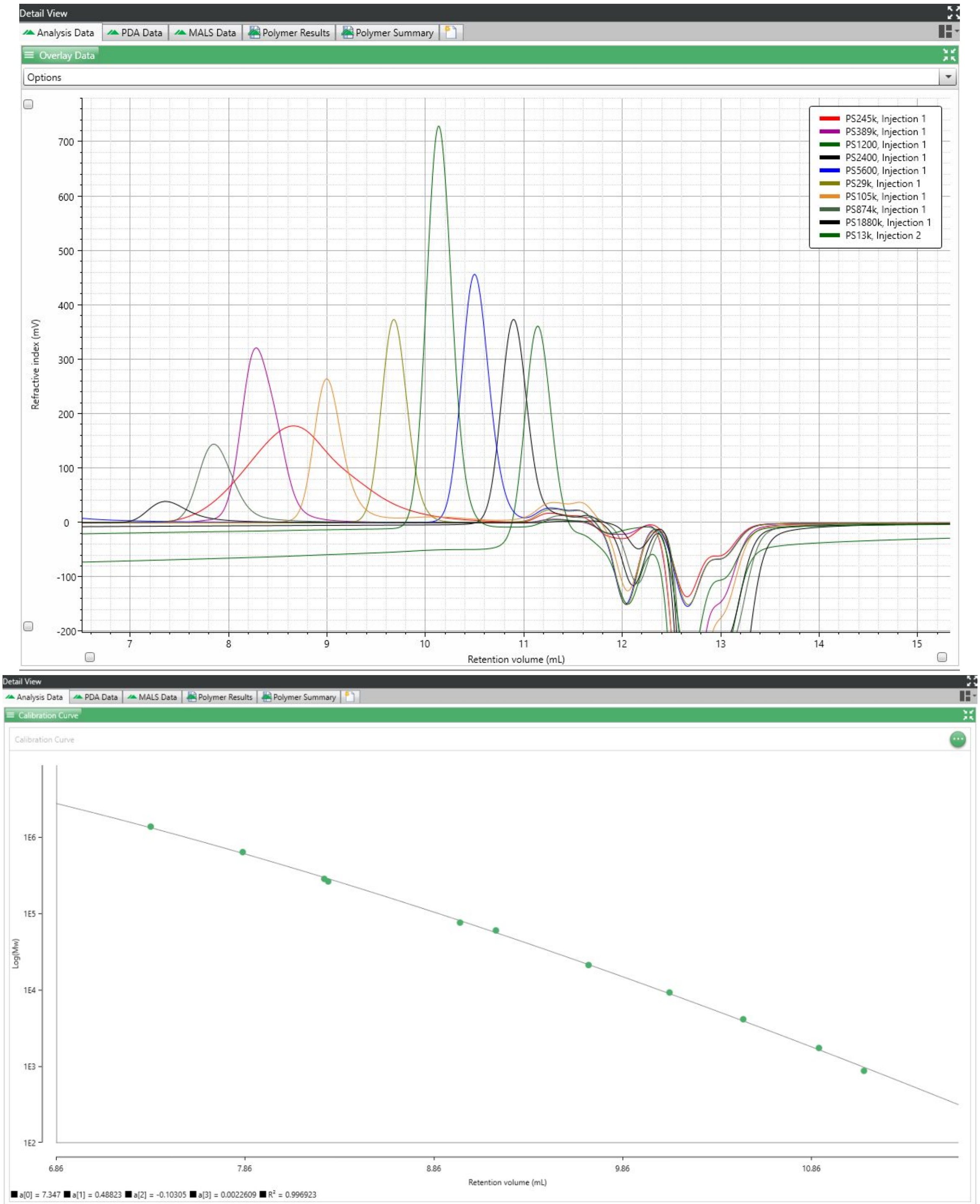

Figure S18. A conventional calibration curve built using a range of different narrow and broad polystyrene standards.

Universal Calibration

A series of well-characterized polystyrene standards with known molecular weights (listed in **Table S4**) were analyzed using the RI and viscometer detector to obtain a universal calibration method which will provide true or accurate molecular weight that is independent standard types. It uses the relationship between hydrodynamic volume or molecular size, molecular weight (MW) and intrinsic viscosity (IV) to show that the calibration curve for polymers of different types can merge onto a single calibration curve.

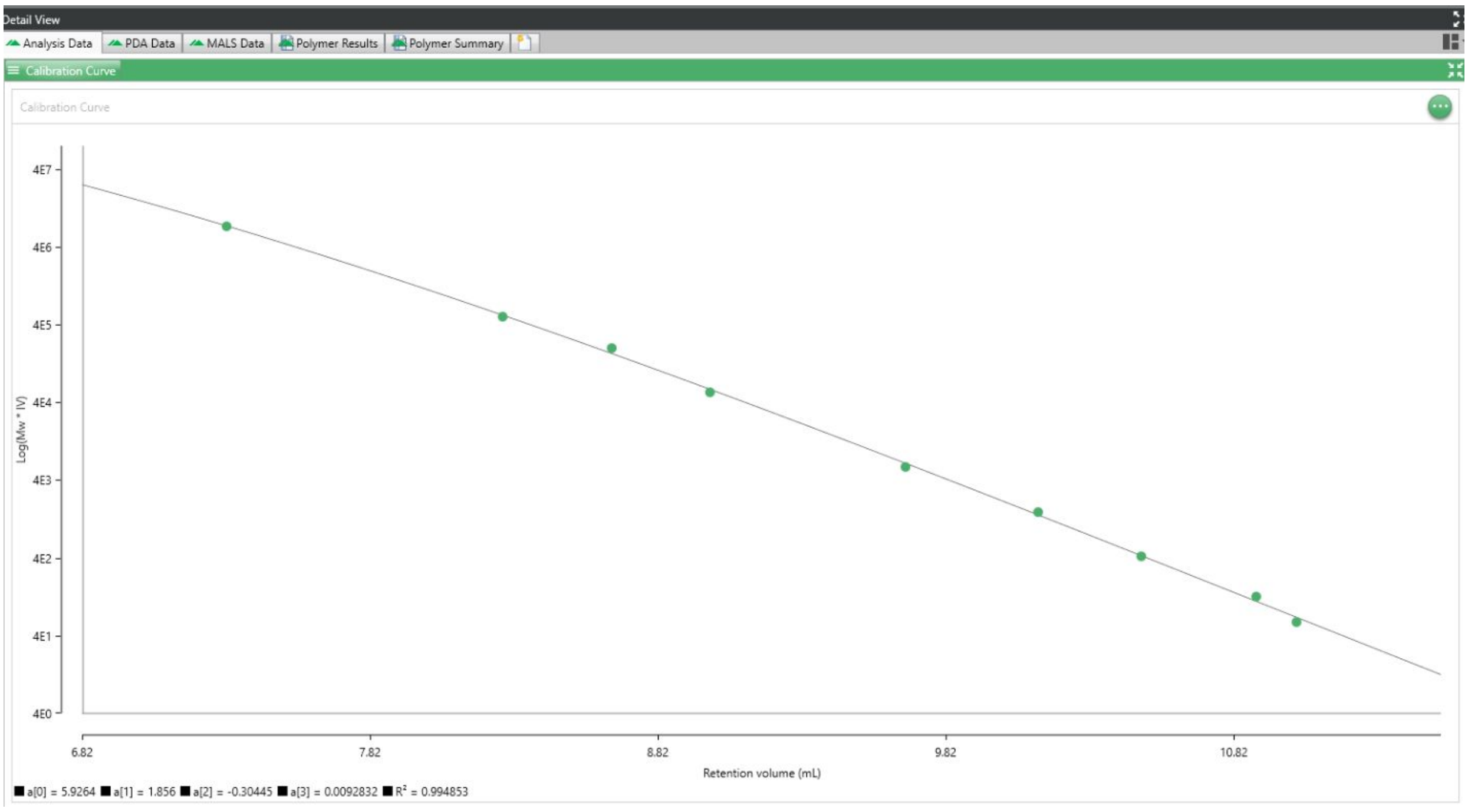

Figure S19. A universal calibration curve built using a range of different narrow and broad polystyrene standards.

Section S7: References

(1) Cazes, J. Current Trends in Gel Permeation Chromatography. Part One: Theory and Equipment. *J. Chem. Educ.* **1970**, *47* (7), A461–A471. DOI: 10.1021/ed047pA461.

(2) Cazes, J. Gel Permeation Chromatography—Part 1. *J. Chem. Educ.* **1966**, *43* (7), A567–A582. DOI: 10.1021/ed043pA567.

(3) Cazes, J. Gel Permeation Chromatography. Part Two. *J. Chem. Educ.* **1966**, *43* (8), A625–A642. DOI:10.1021/ed043pA625.

(4) Cantor, C. R.; Schimmel, P. R. *Biophysical Chemistry, Part II: Techniques for the Study of Biological Structure and Function*; W. H. Freeman: San Francisco, 1980.

(5) Tanford, C. *Physical Chemistry of Macromolecules*; John Wiley & Sons: New York, 1961.
